# The Importance of Hypoxia-Inducible Factors (HIF-1 and HIF-2) for the Pathophysiology of Inflammatory Bowel Disease

**DOI:** 10.3390/ijms21228551

**Published:** 2020-11-13

**Authors:** Evelyn L. Kerber, Claudia Padberg, Nora Koll, Vera Schuetzhold, Joachim Fandrey, Sandra Winning

**Affiliations:** Institute for Physiology, University of Duisburg-Essen, 45147 Essen, Germany; evelyn.hammel@gmx.de (E.L.K.); claudia.padberg@uk-essen.de (C.P.); nora.koll@uni-due.de (N.K.); vera.schuetzhold@uni-due.de (V.S.); sandra.winning@uni-due.de (S.W.)

**Keywords:** IBD, inflammation, HIF-1, HIF-2

## Abstract

(1) Background: Hypoxia is a common feature of inflammation when hypoxia inducible factors (HIFs) adapt cells to conditions of low oxygen tension and inflammation. We studied the role of HIF-1 and HIF-2 in cells of the myeloid lineage in a mouse model of acute colitis. (2) Methods: Mice with and without a conditional knockout for either *Hif-1a* or *Hif-2a* or *Hif-1a* and *Hif-2a* in cells of the myeloid lineage were treated with 2.5% dextran sodium sulfate (DSS) for 6 days to induce an acute colitis. We analyzed the course of inflammation with respect to macroscopic (disease activity index) and microscopic (histology score and immunohistochemical staining of immune cells) parameters and quantified the mRNA expression of cytokines and chemokines in the colon and the mesenteric lymph nodes. (3) Results: A conditional knockout of myeloid *Hif-1a* ameliorated whereas the knockout of *Hif-2a* aggravated murine DSS colitis by increased recruitment of neutrophils to deeper layers of the colon. This led to higher expression of *Il6*, *Ifng*, *Cd11c*, *Cd4*, and *Cd8* in the colon but also induced anti-inflammatory mediators such as *Foxp3* and *Il10.* A conditional knockout of *Hif-1a* and *Hif-2a* did not show any differences compared to wildtype mice. (4) Conclusions: Myeloid HIF-1α and HIF-2α play opposing roles in acute DSS colitis. Thus, not only a cell type specific, but also the isoform specific modulation of HIFs needs to be addressed in attempts to modify HIF for therapeutic purposes.

## 1. Introduction

Chronic inflammatory bowel disease (IBD) is a recurrent inflammatory disease of the gastrointestinal tract. Worldwide, more than 6.8 million people suffer from IBD and the annual prevalence is between 0.5 and 24.5 per 100,000 people every year [1,2]. People affected by IBD also show an increased risk to develop colorectal cancer, depending on the extent and duration of the relapses [3,4]. Although adjustment of lifestyle and drug treatment can reduce the symptoms of IBD, it is not possible to cure many patients [5]. Hypoxia plays a decisive role in inflammatory diseases such as IBD [6,7,8]. The family of hypoxia-inducible factors (HIFs) acts as transcription factors mediating the hypoxic response in cells and tissues. HIFs coordinate a transcriptional program involving a very broad range of physiological functions, including angiogenesis, erythropoiesis, cellular metabolism, autophagy, apoptosis, and other physiological responses. Thus, HIFs ensure optimal functional, metabolic, and vascular adaptation to low O_2_ levels [9]. HIFs are heterodimers consisting of a constitutively expressed HIF-1β subunit and an O_2_-dependent HIF-α subunit [10]. The regulation of HIF-1α and HIF-2α occurs at the post-translational level by hydroxylation of specific proline and asparagine residues. This regulation is to a great extent specific to three PHD isoforms: the prolyl hydroxylase PHD2 predominantly regulates HIF-1α, whereas PHD1 and PHD3 show a higher affinity for HIF-2α [11,12]. Prolyl hydroxylation is O_2_-dependent and initiates proteasomal degradation of the labeled HIF- α protein under high O_2_ conditions [13].

In case of hypoxia, e.g., during tissue inflammation, the PHDs are inactive, which leads to a reduced degradation and nuclear translocation of the HIF-α subunit. In the nucleus, HIF-α forms a heterodimer with HIF-1β and binds to the HREs (hypoxia response elements) of the target gene DNA. The active complex then initiates the transcription of HIF target genes [14].

Higher interstitial pressure and increased oxygen consumption by (mainly immune) cells reduce the oxygen tension in inflamed tissues [6,7,8]. Immune cells migrating into inflamed areas therefore have to adapt their metabolism to hypoxia. Several groups demonstrated the crucial importance of HIFs as regulators of the immune response by targeted HIF-1α and/or HIF-2α knockout in neutrophils, macrophages, dendritic cells (DCs), or T-cells [15,16,17,18,19,20,21]. In our group, a HIF-1α knockout in myeloid cells led to reduced clinical signs of a dextran sodium sulfate (DSS)-induced murine colitis with an increased presence of regulatory T-cells (Tregs) [15]. However, a HIF-1α knockout in DCs showed increased clinical symptoms of DSS-induced colitis in mice with a lower number of Tregs [18]. This demonstrates that HIF-1 can have antagonistic effects in different immune cells. However, at present the function of HIF-2 in immune cells during IBD is still incompletely understood.

Therefore, the aim of this work was not only to decipher the exact influence of HIF-2 on the function of macrophages and neutrophils during IBD, but also to investigate the effect of a loss of both HIF isoforms (HIF-1 and HIF-2) in myeloid cells in experimental murine DSS colitis.

## 2. Results

### 2.1. The Severity of Colon Inflammation is Reduced in Mice Lacking Functional HIF-1α, but Higher in Mice Lacking Functional HIF-2α in Myeloid Cells

The disease activity index (DAI) includes body weight, stool consistency, and the presence of blood in the stool [22]. A daily report of these data allowed following up the severity of colitis. The maximum value of the DAI was 12. The scoring system consisted of three parameters (weight loss, stool consistency, and occurrence of fecal blood) each with a scoring of 0–4 (please methods section for details).

**Figure 1:**
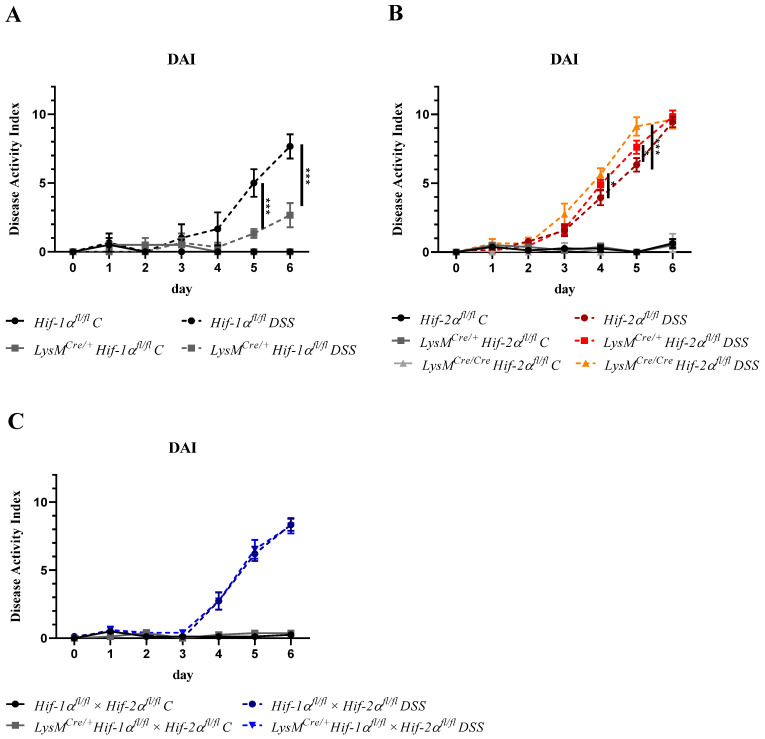
Myeloid HIF-1α knockout ameliorated whereas myeloid HIF-2α knockout aggravated colitis. Daily recording of the disease activity index (DAI) value with separate presentation for the different animal strains with and without dextran sodium sulfate (DSS) treatment. Statistical analysis was performed with a 2-way ANOVA (mean values ± SEM; (**A**): *n*(C) = 2, *n*(DSS) = 3; (**B**): *n*(C) = 3 (*LysM^Cre/Cre^ Hif-2α^fl/fl^*)/8 (*Hif-2α^fl/fl^; LysM^Cre/+^ Hif-2^αfl/fl^*), *n*(DSS) = 8 (*LysM^Cre/Cre^ Hif-2α^fl/fl^*)/18 (*Hif-2α^fl/fl^*; *LysM^Cre/+^ Hif-2α^fl/fl^*); and (**C**): *n*(C) = 7, *n*(DSS) = 14). *: *p* < 0.05; ***: *p* < 0.001.

The control groups treated with drinking water did not show major changes in the DAI during the entire experiment. DSS-treated *Hif-1α^fl/fl^* animals revealed a significantly higher DAI than DSS-treated *LysM^Cre/+^ Hif-1α^fl/fl^* animals from day 5 onwards (Figure 1A). These data indicate that a HIF-1α knockout in myeloid cells ameliorated the progression of a DSS-induced colitis and confirms our previous report (22).

*LysM^Cre/+^ Hif-2α^fl/fl^* and *LysM^Cre/Cre^ Hif-2α^fl/fl^* animals in contrast showed a higher DAI value from day 4 onwards compared to DSS-treated *Hif-2α^fl/fl^* animals (Figure 1B), which correlated with the abundance of Cre recombinase because the Cre/Cre animals with higher knockout efficiency (see Figure S1) also showed a higher DAI. Thus, a myeloid HIF-2α knockout seemed to aggravate the course of the disease.

DSS-treated *LysM^Cre/+^ Hif-1α^fl/fl^* × *Hif-2α^fl/fl^* animals showed a very similar course of colitis compared to the DSS-treated *Hif-1α^fl/fl^* × *Hif-2α^fl/fl^* animals (Figure 1C). Thus, a myeloid double knockout of HIF-1α and HIF-2α compensated for the effects observed with a single knockout and showed no change in the clinical course of colitis compared to mice with both HIFs intact.

### 2.2. Tissue Destruction after DSS Treatment in Mice without Functional HIF-1α is Reduced, but Higher in Mice without Functional HIF-2α in Myeloid Cells

**Figure 2:**
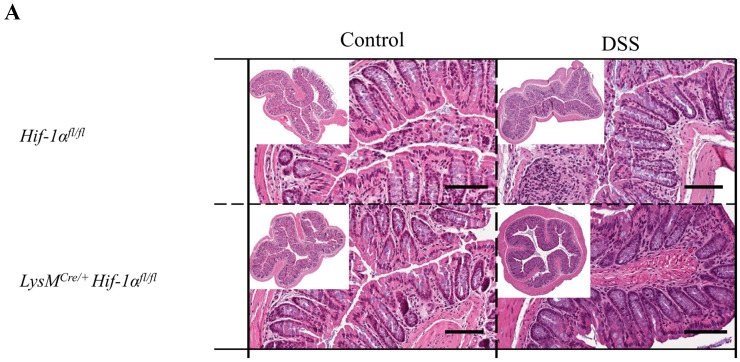

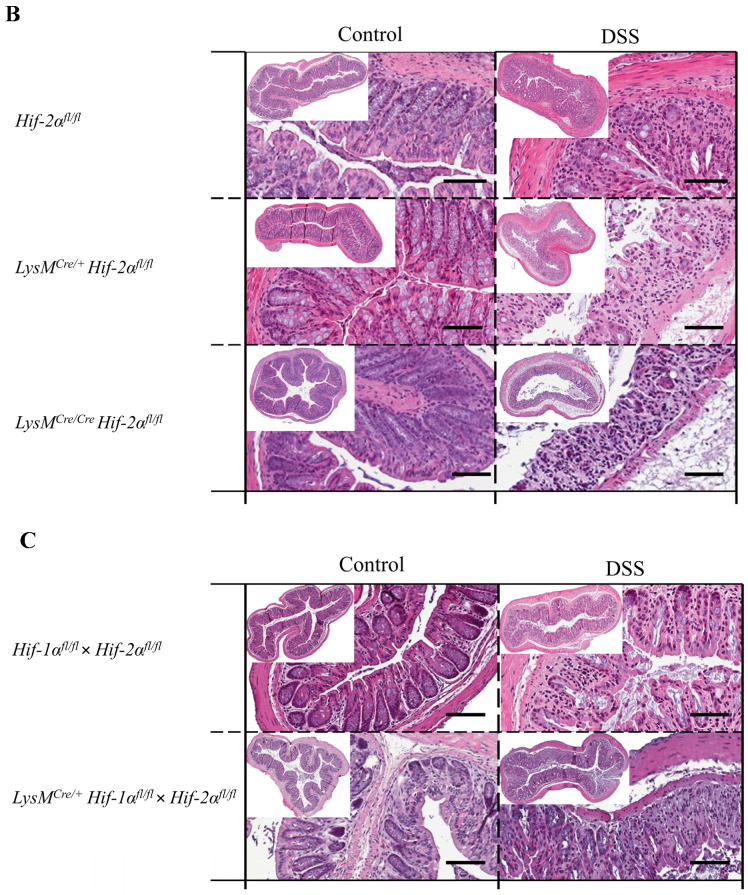
Myeloid knockout of HIF-1α leads to low tissue destruction, while myeloid knockout of HIF-2α increased it. Exemplary presentation of H&E-stained colon tissue sections of (**A**) Hif-1α^fl/fl^, LysM^Cre/+^ Hif-1α^fl/fl^ animals, (**B**) *Hif-2α^fl/fl^*, *LysM^Cre/**+**^ Hif-2α^fl/fl^* and *LysM^Cre/**Cre**^ Hif-2α^fl/fl^* animals, and (**C**) *Hif-1α^fl/fl^* × *Hif-2α^fl/fl^* and LysM^Cre/+^ *Hif-1α^fl/fl^* × *Hif-2α^fl/fl^* animals after DSS treatment and the control group. Tissue sections from DSS-treated *LysM^Cre/**Cre**^ Hif-2α^fl/fl^* animals showed the highest structural damage of tissue. Tissue sections from DSS-treated LysM^Cre/+^ *Hif-1α^fl/fl^* animals showed the least structural damage. A: *n*(Control) = 2, *n*(DSS) = 3; B: *n*(Control) = 3 (*LysM^Cre/**Cre**^ Hif-2α^fl/fl^*)/8 (*Hif-2α^fl/fl^*; *LysM^Cre/**+**^ Hif-2α^fl/fl^*), *n*(DSS) = 8 (*LysM^Cre/**Cre**^ Hif-2α^fl/fl^*)/18 (*Hif-2α^fl/fl^*, *LysM^Cre/**+**^ Hif-2α^fl/fl^*); and C: *n*(Control) = 7, *n*(DSS) = 14. Magnification overview image: 100×; magnification detail image: 200×; and scale bar: 100 µm.

H&E staining of the colon sections was performed for the initial assessment of tissue damage (Figure 2). The control groups showed the typical physiological structure of a healthy colon without any differences between the genotypes. After 6 days of DSS treatment damage of the tissue structure and infiltration of immune cells into the *lamina propria mucosae* were observed in all tissue sections (Figure 2A,B). DSS-treated *LysM^Cre/**Cre**^ Hif-2α^fl/fl^* animals showed the most severe damage of colon tissue structure with almost complete loss of crypts and massive infiltration of immune cells into all tissue layers (lower right panel of Figure 2B). The *tela submucosa* showed a strong edematization and the tunica muscularis was clearly thickened. DSS-treated *LysM^Cre/**+**^ Hif-2α^fl/fl^* animals carrying the less extensive HIF-2α knockout (Figure S1) also showed a distinct loss of tissue structure with infiltration of immune cells up to the tunica muscularis. In addition, destroyed crypts, edematization in the submucosal layer, and a thickened tunica muscularis were observed. However, compared to the tissue sections of the DSS-treated *LysM^Cre/**Cre**^ Hif-2α^fl/fl^* animals, both edematization and destroyed crypts were much less pronounced. Thus, mice with a myeloid HIF-2α knockout revealed an increased damage of colon tissue after DSS-treatment dependent on of the efficiency of the knockout. In analogy to this, the HIF-1α and HIF-2α staining of the colon spreads from the luminal tips of the epithelium in untreated mice towards deeper layers of the tissue indicating that hypoxia and inflammation attained the *tela submucosa* and the tunica muscularis (Figures S2 and S3).

DSS-treated Hif-1α^fl/fl^-, *Hif-2α^fl/fl^*-, and *Hif-1α^fl/fl^* × *Hif-2α^fl/fl^*-, but also LysM^Cre/+^ Hif-1α^fl/fl^ × *Hif-2α^fl/fl^* animals showed a lower damage of the tissue structure with many intact crypts and a lower infiltration of immune cells (Figure 2A,C). The least damage was found in LysM^Cre/+^ Hif-1α^fl/fl^ animals, which exhibited the most intact crypts and a low infiltration of immune cells. These data suggest that a HIF-1α knockout in cells of the myeloid lineage led to less tissue damage in DSS-induced colitis and less disease progression whereas a double knockout of HIF-1α and HIF-2α had no effect on tissue damage during DSS-induced colitis after 6 days.

**Figure 3:**
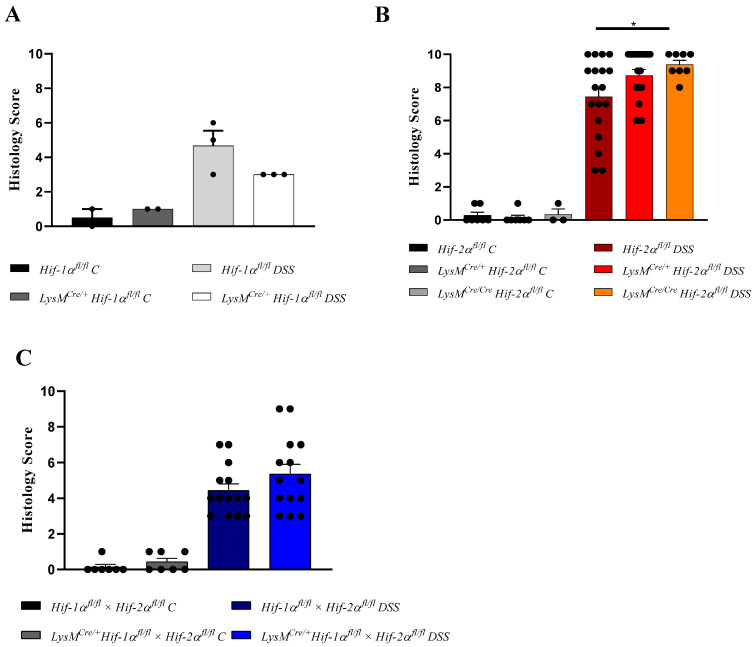
Myeloid knockout of HIF-1α led to a low colon histology score, while myeloid knockout of HIF-2α increased it. H&E-stained sections of the colon of Hif-1α^fl/fl^, LysM^Cre/+^ *Hif-1α^fl/fl^* animals (**A**), *Hif-2α^fl/fl^*, *LysM^Cre/**+**^ Hif-2α^fl/fl^* and *LysM^Cre/**Cre**^ Hif-2α^fl/fl^* animals (**B**), and *Hif-1α^fl/fl^* × *Hif-2α^fl/fl^* and LysM^Cre/+^ *Hif-1α^fl/fl^* × *Hif-2α^fl/fl^* animals (**C**) with and without DSS treatment were evaluated by a histology score (for parameters see Table 2). The DSS-treated *LysM^Cre/**Cre**^ Hif-2α^fl/fl^* animals showed the highest score and DSS-treated LysM^Cre/+^ *Hif-1α^fl/fl^* animals had the lowest score. Statistical analysis was performed with an unpaired *t*-test. Mean values ± SEM; A: *n*(C) = 2, *n*(DSS) = 3; B: *n*(C) = 3 (*LysM^Cre/**Cre**^ Hif-2α^fl/fl^*)/8 (*Hif-2α^fl/fl^*, *LysM^Cre/**+**^ Hif-2α^fl/fl^*), *n*(DSS) = 8 (*LysM^Cre/**Cre**^ Hif-2α^fl/fl^*)/18 (*Hif-2α^fl/fl^*, *LysM^Cre/**+**^ Hif-2α^fl/fl^*); and C: *n*(C) = 7, *n*(DSS) = 14. Each black dot represents the score of a single animal. *: *p* < 0.05.

For a semiquantitative assessment of tissue damage, the H&E-stained tissue sections were used to determine a histology score with a maximum value of 10 (taking the infiltration of immune cells, destruction of crypts, and the extent of damage into account (please methods section for details). The control groups showed a score of almost 0 (0 or 1). DSS-treated Hif-1α^fl/fl^, *Hif-1α^fl/fl^* × *Hif-2α^fl/fl^* and LysM^Cre/+^ *Hif-1α^fl/fl^* × *Hif-2α^fl/fl^* animals showed score values between 3 and 8 (Figure 3A,C). *Hif-2α^fl/fl^* animals showed a higher variation in score values between 3 and 10 (Figure 3B). *LysM^Cre/**+**^ Hif-2α^fl/fl^* animals showed score values between 6 and 10. The highest score between 8 and 10 and thus the most severe tissue damage was observed in *LysM^Cre/**Cre**^ Hif-2α^fl/fl^* animals. LysM^Cre/+^ *Hif-1α^fl/fl^* animals showed relatively limited damage of colonic tissue structure with a consistent score of 3 (Figure 3A). This supports our previous findings [15] that a knockout of HIF-1α in myeloid cells leads to less tissue damage, a knockout of HIF-2α results in increased tissue damage and inflammation and a double knockout of HIF-1α and HIF-2α does not alter tissue damage compared to LysMCre negative animals in DSS-induced colitis. Further analysis of *Hif-1α^fl/fl^* and LysM^Cre/**+**^ *Hif-1α^fl/fl^* has already been published by our group [15], thus herein we will focus on the observations in *Hif-2α^fl/fl^* and *Hif-1α^fl/fl^* × *Hif-2α^fl/fl^* mice.

### 2.3. Tissue Destruction Reduced the Mucin Production in the Colon

**Figure 4:**
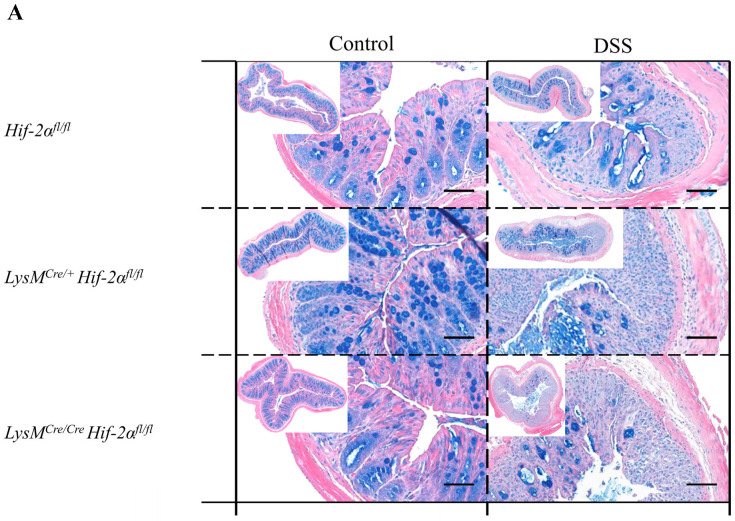

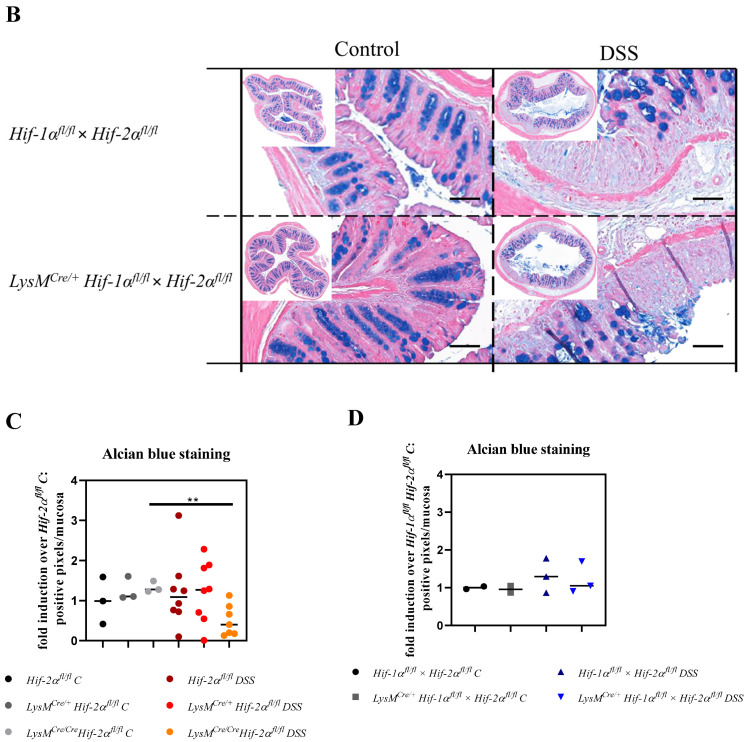
Reduced mucin production after tissue destruction. Exemplary presentation of alcian blue-stained colon tissue sections of (**A**) *Hif-2α^fl/fl^*, *LysM^Cre/+^ Hif-2α^fl/fl^* and *LysM^Cre/**Cre**^ Hif-2α^fl/fl^* animals and (**B**) *Hif-1α^fl/fl^* × *Hif-2α^fl/fl^* and LysM^Cre/+^ *Hif-1α^fl/fl^* × *Hif-2α^fl/fl^* animals with and without DSS treatment and quantification of histological data (**C**,**D**). No positive alcian blue staining was observed in damaged colon areas. Statistical analysis was performed with an unpaired *t*-test; (**A**): *n*(Control) = 3 (*LysM^Cre/**Cre**^ Hif-2α^fl/fl^*)/5 (*Hif-2α^fl/fl^*, *LysM^Cre/**+**^ Hif-2α^fl/fl^*), *n*(DSS) = 8 (*LysM^Cre/**Cre**^ Hif-2α^fl/fl^*)/12 (*Hif-2α^fl/fl^*, *LysM^Cre/**+**^ Hif-2α^fl/fl^*), (**B**): *n*(Control) = 2, *n*(DSS) = 3. counterstaining: eosin. Magnification overview image: 100×; magnification detail image: 200×; and scale bar: 100 µm, (**C**,**D**): every indicated spot depicts the quantitative analysis of a complete cross section of one colon. **: *p* < 0.01.

The protective barrier of the gastrointestinal tract is supported by mucin production. All mucopolysaccharides and acetic acid mucins were therefore analyzed by means of an alcian blue staining. The animals of the control groups showed small, circular structures stained by alcian blue (Figure 4). In the basal half of the *tunica mucosa* these structures were close together and corresponded with the position of the Goblet cells. In the luminal half of the *tunica mucosa* large, round structures were occasionally observed. No clear differences between the different genotypes were detected (Figure 4C,D). The colon tissue sections of the DSS-treated animals showed mainly large, round structures. In damaged areas of the colon tissue, no staining with alcian blue was visible and the quantitative analysis revealed significantly less alcian blue expression in DSS treated *LysM^Cre/**Cre**^ Hif-2α^fl/fl^* animals (Figure 4C). Additionally, the colon tissue sections of *Hif-2α^fl/fl^*, *LysM^Cre/**+**^ Hif-2α^fl/fl^* and *LysM^Cre/**Cre**^ Hif-2α^fl/fl^* animals showed many positive cell nuclei stained with alcian blue. These were hardly detectable in the tissue sections of *Hif-1α^fl/fl^* × *Hif-2α^fl/fl^* and LysM^Cre/+^ *Hif-1α^fl/fl^* × *Hif-2α^fl/fl^* animals (Figure 4B), which also exhibited more large, round alcian blue stained structures in the *tunica mucosa* and changes in overall alcian blue quantification. Thus, in animals with a HIF-2α knockout the lowest alcian blue staining was found due to the highest tissue damage after DSS treatment (Figure 4A,C). This indicates that extensive tissue damage reduced the production of mucins in the colon.

### 2.4. Macrophage Migration towards the Inflamed Colon is not Altered by Myeloid HIF-2α Knockout

Due to the fact that macrophages played a crucial role in the DSS model of LysM^Cre/+^ Hif-1α^fl/fl^- animals [15] and that macrophages are the cells carrying the knockout analyzed herein, the F4/80 protein was detected in colon tissue sections with the help of a specific antibody.

**Figure 5:**
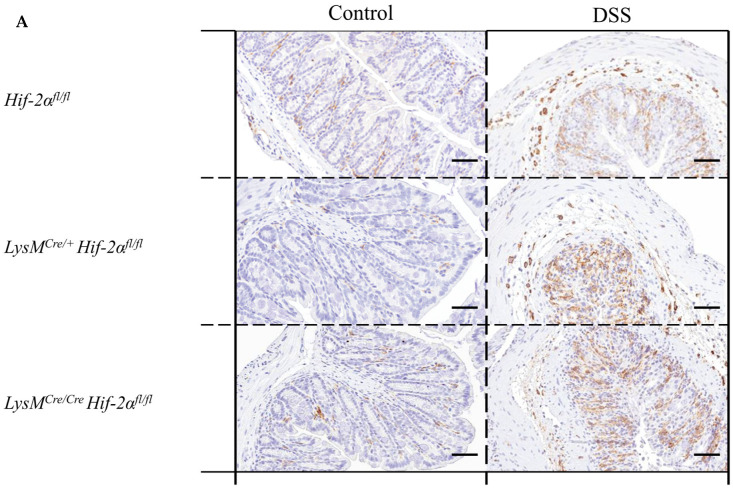

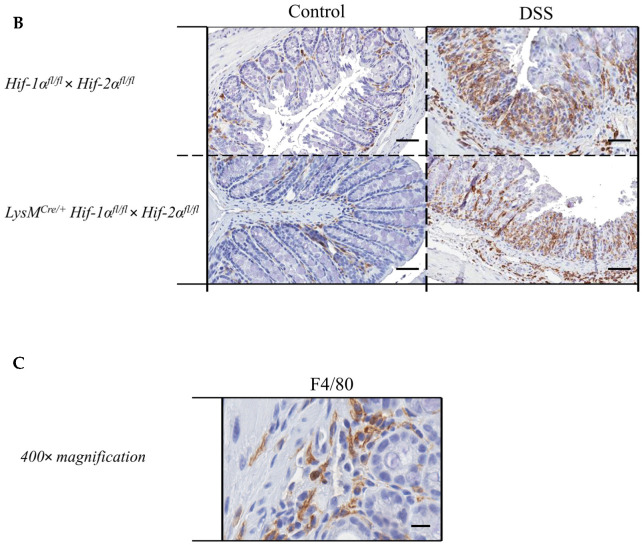
Macrophages without functional HIF-2α showed a similar migration pattern compared to wildtype cells. Exemplary presentation of F4/80-stained colon tissue sections of (**A**) *Hif-2α^fl/fl^*, *LysM^Cre/**+**^ Hif-2α^fl/fl^* and *LysM^Cre/**Cre**^ Hif-2α^fl/fl^* animals, and (**B**) *Hif-1α^fl/fl^* × *Hif-2α^fl/fl^* and LysM^Cre/+^ *Hif-1α^fl/fl^* × *Hif-2α^fl/fl^* animals with and without DSS treatment and an exemplary high-resolution image that shows the specificity of the staining (**C**). After DSS treatment, increased numbers of F4/80-positive cells were observed in the *lamina propria mucosae* and *tela submucosa* in all tissue sections. (**A**): *n*(Control) = 3 (*LysM^Cre/**Cre**^ Hif-2α^fl/fl^*)/5 (*Hif-2α^fl/fl^*, *LysM^Cre/**+**^ Hif-2α^fl/fl^*), *n*(DSS) = 8 (*LysM^Cre/**Cre**^ Hif-2α^fl/fl^*)/12 (*Hif-2α^fl/fl^*, *LysM^Cre/**+**^ Hif-2α^fl/fl^*); (B): *n*(Control) = 7, *n*(DSS) = 14. (**A**) and (**B**): magnification 200×, scale bar: 100 µm; (**C**): magnification 400×, scale bar: 50 µm.

The control groups showed only a few F4/80-positive cells in the *lamina propria mucosae* and sporadically in the *tela submucosa*. In the tissue sections of the DSS-treated animals, there was a clear increase in the number of F4/80-positive cells in the *lamina propria mucosae* and in the *tela submucosa*. However, there were no clear differences between the different genotypes within the DSS-treated group (Figure 5A,B). This result indicates that HIF-2α might play a minor role for the migration of macrophages.

### 2.5. Mice without Functional HIF-2α in Myeloid Cells Recruit more Neutrophils towards the Gut Lumen

Due to the fact that neutrophils played a crucial role in inflammation [23] and are the cells carrying the knockout analyzed herein, the MPO protein was detected in colon tissue sections with the help of a specific antibody.

**Figure 6:**
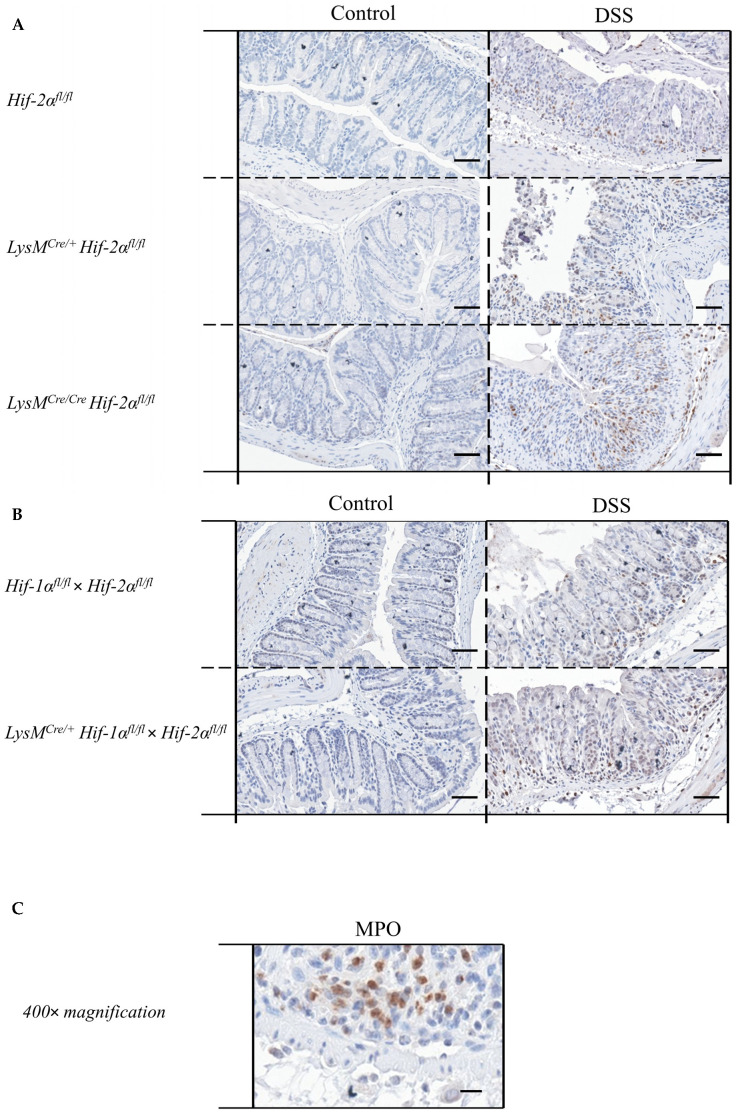

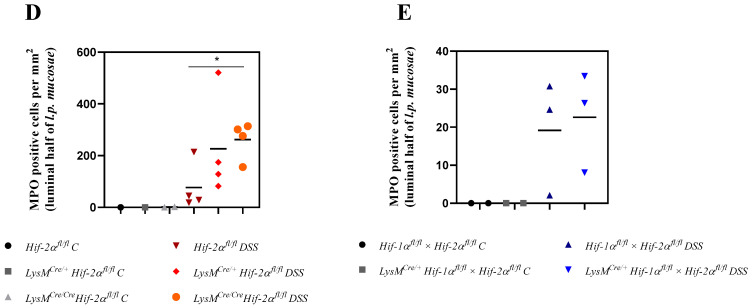
Neutrophils without functional HIF-2α showed a higher migration pattern compared to wildtype cells. Exemplary presentation of MPO-stained colon tissue sections of (**A**) *Hif-2α^fl/fl^*, *LysM^Cre/**+**^ Hif-2α^fl/fl^* and *LysM^Cre/**Cre**^ Hif-2α^fl/fl^* animals, and (**B**) *Hif-1α^fl/fl^* × *Hif-2α^fl/fl^* and LysM^Cre/+^ *Hif-1α^fl/fl^* × *Hif-2α^fl/fl^* animals with and without DSS treatment and an exemplary high-resolution image that shows the specificity of the staining (**C**). Quantification of histological data (**D**,**E**) show no significant differences. Statistical analysis was performed with an unpaired *t*-test; (**A**): *n*(Control) = 3 (*LysM^Cre/**Cre**^ Hif-2α^fl/fl^*)/8 (*Hif-2α^fl/fl^*, *LysM^Cre/**+**^ Hif-2α^fl/fl^*), *n*(DSS) = 8 (*LysM^Cre/**Cre**^ Hif-2α^fl/fl^*)/18 (*Hif-2α^fl/fl^*, *LysM^Cre/**+**^ Hif-2α^fl/fl^*); (**B**): *n*(Control) = 7, n(DSS) = 14. (**A**) and (**B**): magnification 200×, scale bar: 100 µm; (**C**): magnification 400×, scale bar: 50 µm. (**D**,**E**): every indicated spot depicts the quantitative analysis of 4 sections (400× magnification) per colon. *: *p* < 0.05.

The control groups showed no MPO-positive cells in the colon tissue sections. In the tissue sections of the DSS-treated animals, there was a clear increase in the number of MPO-positive cells (Figure 6A,B). DSS-treated *Hif-2α^fl/fl^*, *Hif-1α^fl/fl^* × *Hif-2α^fl/fl^* and LysM^Cre/+^ *Hif-1α^fl/fl^* × *Hif-2α^fl/fl^* animals showed MPO-positive cells especially in the *tela submucosa* and in the basal part of the *lamina propria mucosae*. DSS- treated *LysM^Cre/**+**^ Hif-2α^fl/fl^* und especially DSS-treated *LysM^Cre/**Cre**^ Hif-2α^fl/fl^* animals showed MPO-positive cells also in the *tela submucosa* but also increased at the top of the *lamina propria mucosae* (Figure 6D). This result indicates that HIF-2α might play a role for the invasion of neutrophils.

### 2.6. Mice without Functional HIF-2α in Myeloid Cells Recruit more Neutrophils, CD4^+^, and CD8a^+^ T-Cells and Tregs towards the Inflamed Colon

Immune cells have to raise an adequate immune response during massive antigen exposure and play an important role in IBD [24,25]. For quantitative detection of immune cells in the colon, specific markers were detected by qPCR in whole colon RNA samples. All data were depicted as the fold increase over wildtype control (also see methods section). For this reason the controls were not explicitly shown in the figures and are found in the (supplements Tables S1–S3).

**Figure 7:**
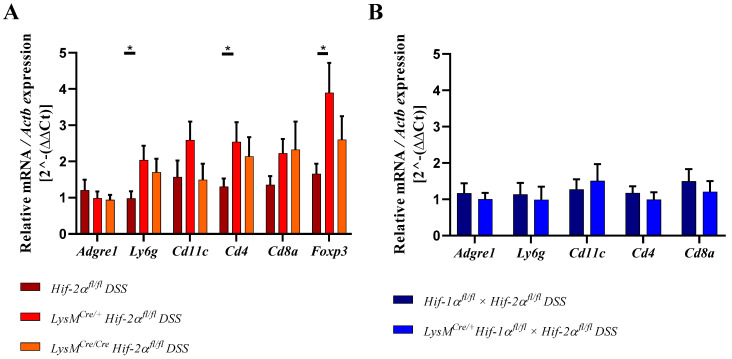
Myeloid knockout of HIF-2α increased the numbers of neutrophils and T-cells in inflamed colon tissue. The expression of *Adgre1*, *Ly6g*, *Cd11c*, *Cd4*, *Cd8a*, and *Foxp3* were quantitatively determined by qPCR in the colonic tissue of DSS-treated animals. After 6 days of DSS treatment an increased expression of *Ly6g*, *Cd4*, *Cd8a*, and *Foxp3* were observed in RNA samples from DSS-treated *LysM^Cre/**+**^ Hif-2α^fl/fl^* and LysM^Cre/**Cre**^ Hif-2α^fl/f^ animals. Statistical analysis was performed with an unpaired *t*-test (mean values ± SEM; (**A**): *n* = 6 (LysM^Cre/**Cre**^ Hif-2α^fl/f^)/15 (*Hif-2α^fl/fl^*, *LysM^Cre/**+**^ Hif-2α^fl/fl^*); (**B**): *n* = 14). All data were normalized to the untreated wildtype control. Gene expression of untreated animals did not differ markedly from the respective wildtype control and is depicted in suppl. Figure S4. *: *p* < 0.05.

The specific macrophage marker F4/80 was used for the identification of macrophages. In the mouse it is encoded by the *Adgre1* gene. We observed no altered expression of *Adgre1* neither between the genotypes nor within the groups (Figure 7). These data further indicate that HIF-2 has no influence on macrophage migration.

To characterize the numbers of neutrophils we quantified the *Ly6g* mRNA of the whole colon by qPCR [26]. After DSS treatment *LysM^Cre/**+**^ Hif-2α^fl/fl^* animals showed a significantly increased *Ly6g* expression compared to DSS-treated *Hif-2α^fl/fl^* animals (Figure 7). An increased *Ly6g* expression was also observed in DSS-treated *LysM^Cre/**Cre**^ Hif-2α^fl/fl^* animals. In DSS-treated *Hif-2α^fl/fl^*, *Hif-2α^fl/fl^* × *Hif-1α^fl/fl^* and *LysM^Cre/+^ Hif-2α^fl/fl^* × *Hif-1α^fl/fl^* animals and untreated animals there were no differences in *Ly6g* expression. Therefore, HIF-2 might influence neutrophil migration towards inflamed tissue (Figure 7A) whereas neutrophil migration was unaffected when both HIF isoforms were missing (Figure 7B).

We also analyzed the numbers of dendritic cells in the colon by detection of the *Cd11c* mRNA (Figure 7). In samples of the DSS-treated *LysM^Cre/**+**^ Hif-2α^fl/fl^* animals a tendency towards an increased *Cd11c* expression was observed. In all other genotypes, but also comparing non-treated and DSS-treated animals, there were no differences in the *Cd11c* expression. These data indicate that HIF-2 had no clear influence on the abundance of dendritic cells after 6 days of DSS treatment.

**Figure 8:**
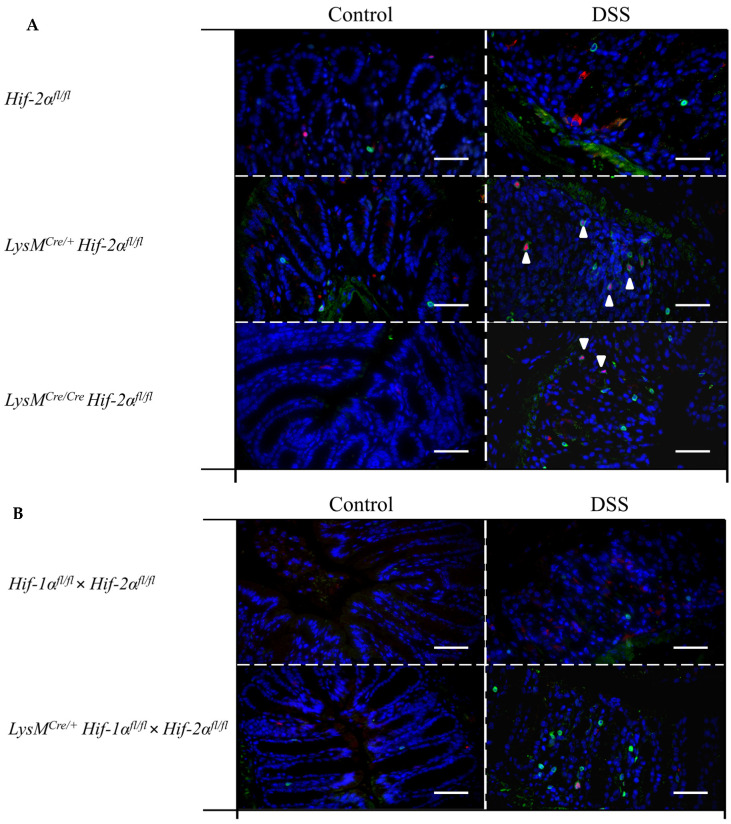
Myeloid knockout of HIF-2α increased the numbers of T-cells and Treg cells in inflamed colon tissue. Exemplary presentation of CD3- (green, surface staining) and FoxP3- (red, nuclear staining) stained colon tissue sections of (**A**) *Hif-2α^fl/fl^*, *LysM^Cre/+^ Hif-2α^fl/fl^* and *LysM^Cre/**Cre**^ Hif-2α^fl/fl^* animals, and (**B**) *Hif-1α^fl/fl^* × *Hif-2α^fl/fl^* and LysM^Cre/+^ *Hif-1α^fl/fl^* × *Hif-2α^fl/fl^* animals with and without DSS treatment. (**A**,**B**): *n*(Control) = 2; *n*(DSS) = 4 (magnification 200×, scale bar: 100 µm). Marked cells (arrows) are double-positive for CD3 and FoxP3.

To quantitate the contribution of cells of the adaptive immune system *Cd4* and *Cd8a* mRNA were detected in whole colon samples using qPCR (Figure 7A,B). CD4 serves as a marker for T-helper cells and CD8a is mainly expressed by cytotoxic T-cells [27]. Samples from DSS-treated *LysM^Cre/**+**^ Hif-2α^fl/fl^* and *LysM^Cre/**Cre**^ Hif-2α^fl/fl^* animals showed higher *Cd4* and *Cd8a* expression compared to samples from DSS-treated *Hif-2α^fl/fl^* animals or the control group (Figure 7). Samples from DSS-treated *Hif-2α^fl/fl^* animals were not different from the control group. Samples from DSS-treated *Hif-1α^fl/fl^* × Hif- 2α^fl/fl^ and *LysM^Cre/+^ Hif-2α^fl/fl^* × *Hif-1α^fl/fl^* animals showed no differences in *Cd4* and *Cd8a* expression (Figure 7B). Additionally *Foxp3* was observed in *Hif-2α^fl/fl^*, *LysM^Cre/**+**^ Hif-2α^fl/fl^* and *LysM^Cre/**Cre**^ Hif-2α^fl/fl^* animals. FOXP3 serves as a marker for Tregs [28,29]. After DSS treatment, samples from *LysM^Cre/**+**^ Hif-2α^fl/fl^* and *LysM^Cre/**Cre**^ Hif-2α^fl/fl^* animals showed an increased *Foxp3* expression compared to samples from DSS-treated *Hif-2α^fl/fl^* animals (Figure 7A). To investigate whether these changes in mRNA expression also correlate with the recruitment of T cells to the inflamed colon we stained colon sections of control and DSS treated *Hif-2α^fl/fl^*, *LysM^Cre/**+**^ Hif-2α^fl/fl^* and *LysM^Cre/**Cre**^ Hif-2α^fl/fl^* (Figure 8A) and *Hif-1α^fl/fl^* × Hif- 2α^fl/fl^ and *LysM^Cre/+^ Hif-2α^fl/fl^* × *Hif-1α^fl/fl^* (Figure 8B) animals for the T cell marker CD3 (green) and for FoxP3 (red). Herein, we found more CD3 and FoxP3 double-positive cells in *LysM^Cre/**+**^ Hif-2α^fl/fl^* and *LysM^Cre/**Cre**^ Hif-2α^fl/fl^* compared to *Hif-2α^fl/fl^* after DSS treatment. DSS treatment induced the numbers of CD3 positive cells in *Hif-1α^fl/fl^* × Hif- 2α^fl/fl^ and *LysM^Cre/+^ Hif-2α^fl/fl^* × *Hif-1α^fl/fl^* animals as well but we found less double-positive Tregs here. In DSS-treated *Hif-2α^fl/fl^* animals, no increased *Foxp3* expression was observed compared to untreated animals (Figure 7A). These data indicate that HIF-2 in myeloid cells is likely to influence the CD4^+^ and CD8a^+^ T-cell and Treg abundancy in the inflamed colon.

### 2.7. Mice without Functional HIF-2α in Myeloid Cells Induce Migration of Lymphocytes towards the Gut 

The infiltration of adaptive immune cells occurs most likely from the surrounding lymph nodes. These structures belong to the secondary lymphatic organs and are activated by the presence of bacteria and viruses in the lymph fluid, which results in lymph node swelling and later immune cell evasion towards a chemokine gradient [30,31].

**Figure 9:**
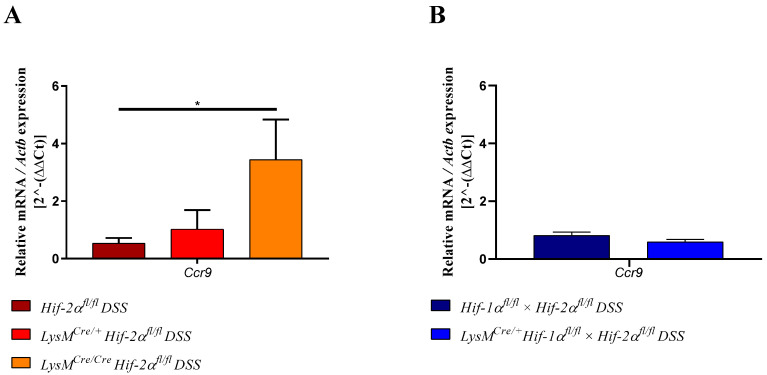
Myeloid knockout of HIF-2α induced the migration of lymphocytes towards the gut. The expression of *Ccr9* was quantitatively determined by qPCR in mesenteric lymph nodes of DSS-treated animals. After 6 days of DSS treatment a significantly increased expression of *Ccr9* was observed in RNA samples from DSS- *LysM^Cre/**Cre**^ Hif-2α^fl/fl^* animals. Statistical analysis was performed with an unpaired *t*-test (mean values ± SEM; (**A**): *n* = 7 (*LysM^Cre/**Cre**^ Hif-2α^fl/fl^*)/10 (*Hif-2α^fl/fl^*; *LysM^Cre/**+**^ Hif-2α^fl/fl^*); (**B**) *n* = 7/8). All data were normalized to the untreated wildtype control. Gene expression of untreated animals did not differ markedly from the respective wildtype control and is depicted in suppl. Figure S5. *: *p* < 0.05.

CCR9 is expressed by dendritic cells but also by lymphocytes and promotes the migration of these cells into the gut [32,33]. RNA samples from lymph nodes of DSS-treated *LysM^Cre/**Cre**^ Hif-2α^fl/fl^* animals showed a significantly higher expression of *Ccr9* than samples from *Hif-2α^fl/fl^* animals (Figure 9A). Samples from DSS-treated *LysM^Cre/**+**^ Hif-2α^fl/fl^* animals exhibited only a slightly increased expression of *Ccr9* compared to samples from *Hif-2α^fl/fl^* animals. Lymph node RNA samples from DSS-treated *Hif-1α^fl/fl^* × *Hif-2α^fl/fl^* and *LysM^Cre/+^* *Hif-1α^fl/fl^* × *Hif-2α^fl/fl^* animals showed no differences (Figure 9B). This indicates that the high inflammation in HIF-2α knockout animals seemed to induce a higher migration of lymphocytes towards the gut.

### 2.8. HIF-2α Loss in Myeloid Cells induced High Pro- and Anti-Inflammatory Gene Expressions in the Inflamed Gut

The ratio of pro- and anti-inflammatory cytokines plays a major role in balancing the inflammation. For this reason, different cytokines were quantitatively detected by qPCR in RNA samples from the whole colon.

**Figure 10:**
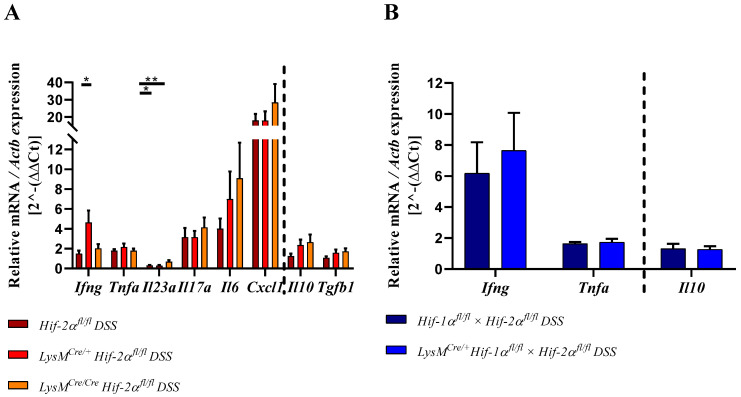

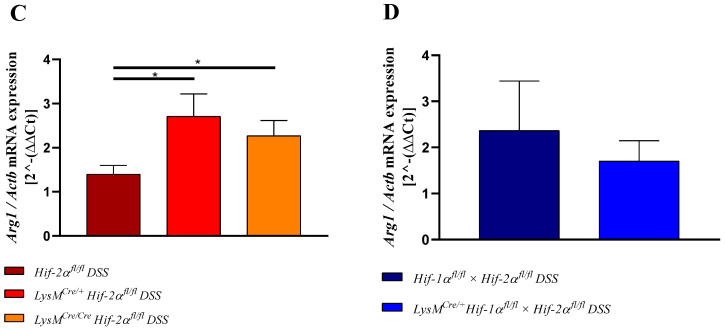
Myeloid knockout of HIF-2α induces both the pro- and the anti-inflammatory cytokine response during inflammation. Figure 10 (**A**,**B**) shows the expression of pro- (left of the dotted line) and antiinflammatory (right of the dotted line) cytokines. Cytokines were quantified by qPCR in the colonic tissue of DSS-treated animals. Figure 10 (**C**,**D**) shows the expression of *Arg1* quantified by qPCR. Statistical analysis was performed with an unpaired *t*-test (mean values ± SEM; A: *n* =6 (*LysM^Cre/**Cre**^ Hif-2α^fl/fl^*)/15 (*Hif-2α^fl/fl^*; *LysM^Cre/**+**^ Hif-2α^fl/fl^*); B: *n* = 14). All data were normalized to the untreated wildtype control. Gene expression of untreated animals did not differ markedly from the respective wildtype control and is depicted in suppl. Figure S6. *: *p* < 0.05; **: *p* < 0.01.

IFNγ, TNFα, IL23, IL17a, IL6, and CXCL1 belong to the proinflammatory cytokines and are expressed by immune cells [34,35,36]. In samples from DSS-treated *LysM^Cre/**+**^ Hif-2α^fl/fl^* animals increased *Ifng* expression was observed compared to samples from DSS-treated *Hif-2α^fl/fl^* and *LysM^Cre/**Cre**^ Hif-2α^fl/fl^* animals and samples from the control group (Figure 10). *Tnfa* and *Il17a* expression were increased in all DSS-treated animals without significant differences between the groups. Additionally, *Cxcl1* was increased in samples from DSS-treated *Hif-2α^fl/fl^*, *LysM^Cre/**+**^ Hif-2α^fl/fl^*, and *LysM^Cre/**Cre**^ Hif-2α^fl/fl^* animals compared to the control group (Figure 10). No difference in expression was observed between samples from DSS-treated *Hif-2α^fl/fl^* and *LysM^Cre/**+**^ Hif-2α^fl/fl^* animals, whereas samples from DSS-treated *LysM^Cre/**Cre**^ Hif-2α^fl/fl^* animals showed an additional increase in their *Cxcl1* expression. *Il6* expression was stepwise increased in samples from DSS-treated *Hif-2α^fl/fl^*, *LysM^Cre/**+**^ Hif-2α^fl/fl^*, and *LysM^Cre/**Cre**^ Hif-2α^fl/fl^* animals (Figure 10). In contrast, in samples from DSS-treated *Hif-2α^fl/fl^* and *LysM^Cre/**+**^ Hif-2α^fl/fl^* animals we observed a significantly decreased *Il23a* expression compared to samples from the control group and to samples from DSS-treated *LysM^Cre/**Cre**^ Hif-2α^fl/fl^* animals. In samples of DSS-treated *LysM^Cre/**Cre**^ Hif-2α^fl/fl^* animals, we observed a lower expression of *Il23a* but in contrast to the expression in the latter groups it was no longer significantly different from the control group. RNA samples from DSS-treated *Hif-1α^fl/fl^* × *Hif-2α^fl/fl^* and LysM^Cre/+^ *Hif-1α^fl/fl^* × *Hif-2α^fl/fl^* animals also showed an increased *Ifng* expression with a higher SEM and a slightly increased *Tnfa* expression.

Taken these data together, animals with a HIF-2α deletion in myeloid cells showed a higher expression of proinflammatory cytokines.

The anti-inflammatory cytokine IL10 is mainly produced by T-cells and can inhibit proinflammatory responses of the innate and the adaptive immune system [37] whereas TGF-β1 most potently inhibits Th1 answers but can also induce the generation of Th17 together with IL6. In Figure 10A (right of broken line), no differences were observed between the *Il10* and *Tgfb1* expression in the colon of DSS-treated *Hif-2α^fl/fl^* animals and the respective control group. Samples from DSS-treated *LysM^Cre/**+**^ Hif-2α^fl/fl^* and *LysM^Cre/**Cre**^ Hif-2α^fl/fl^* animals in contrast showed an increased expression of *Il10* and a higher expression of *Tgfb1* compared to either the respective control group or to samples of DSS-treated *Hif-2α^fl/fl^* animals. In the samples from DSS-treated *Hif-1α^fl/fl^* × *Hif-2α^fl/fl^* and LysM^Cre/+^ *Hif-1α^fl/fl^* × *Hif-2α^fl/fl^* animals no differences in *Il10* expression were observed compared to samples from non-treated animals (Figure 10B; right of broken line). The mRNA of Arginase-1 (arg-1, Figure 10 C,D), an enzyme that has been described as target gene of HIF-2 was surprisingly induced instead of reduced in DSS treated *LysM^Cre/+^ Hif-2α^fl/fl^* and *LysM^Cre/Cre^ Hif-2α^fl/fl^* animals and unaltered in DSS treated *LysM^Cre/+^ Hif-1α^fl/fl^* × *Hif-2α^fl/fl^* mice. Summarizing these data, a higher colon inflammation caused by the myeloid HIF-2 knockout was likely to demand a higher anti-inflammatory response to compensate the otherwise overwhelming inflammation.

## 3. Discussion

Previous studies have shown that HIF-1 plays a predominantly anti-inflammatory role in T-cells, DCs, and epithelial cells and a mainly proinflammatory role in macrophages during IBD [15,18,38,39]. In this study, we observed an inflammation-promoting role of HIF-1 in myeloid cells during DSS-induced colitis (Figure 1 and Figure 2) and confirmed the results of Bäcker et al. [15]. In addition, Cramer et al. (2003) had already observed a diminished inflammatory response in animals with a myeloid HIF-1α knockout in a model of acute skin inflammation [17]. However, a myeloid HIF-2α knockout appears to result in an opposite phenotype in DSS-induced colitis (Figure 1 and Figure 2). Thus, HIF-2 in myeloid cells may have a protective role during IBD. Lin et al. (2018) also observed an increased inflammation in the model of DSS-induced colitis in mice with a myeloid HIF-2α knockout and pointed out the protective role of HIF-2 in myeloid cells during IBD [40]. However, Kim et al. (2018) found no differences in the degree of inflammation between mice with and without HIF-2α in neutrophils (with a *Hif-2a* expression driven by the hMRP8Cre gene promoter) [41]. Reasons for these controversial results could lie in the usage of different Cre promoters and in differing DSS protocols. Kim and coworkers applied 5% DSS for 5 days; this rather high DSS dose might have masked subtle differences between the genotypes due to a severe inflammation of the colon and the extensive tissue damage. Xue et al. (2013) also investigated the role of HIF-2 in the pathophysiology of IBD, but his group used animals with a HIF-2α knockout in the epithelial cells of the intestine [42]. After 7 days of DSS (3%) treatment, a milder course of colitis was observed in the HIF-2α knockouts. This is most likely due to the use of a HIF-2α knockout in a different type of cell. Several groups have already published diverging roles of HIF-1α in different cell types in DSS colitis. A milder course of colitis was observed with a HIF-1α knockout in myeloid cells [15,41] and a stronger course of disease with a HIF-1α knockout in DCs, T-cells, and intestinal epithelial cells [18,38,39]. Thus, in IBD, HIF-1, and HIF-2 play different roles in different cell types. This contrasting role is particularly evident in the *LysM^Cre/+^ Hif-1α^fl/fl^* × *Hif-2α^fl/fl^* animals: the absence of both HIF-1 and HIF-2 in myeloid cells does not affect the symptoms of DSS colitis at all (Figure 1 and Figure 2). This again emphasizes an antagonistic role of HIF-1 and HIF-2 in myeloid cells in IBD. To establish a double knockout of HIF-1α and HIF-2α in myeloid cells, Lin et al. (2018) used LysM^Cre/+^ Arnt^fl/fl^ animals that showed a higher weight loss, a higher DAI and more extended colon damage after DSS treatment compared to Cre-negative animals [40]. However, it is well accepted that ARNT does not only dimerize with HIF-1α or HIF-2α (under hypoxic conditions), but also interacts with the aryl hydrocarbon receptor (AHR), with SIM1, SIM2 (single-minded protein), or ARNT itself, thereby regulating other genes than HIFs [43]. For this reason, the double knockout model used in this study seems to be a more precise model to understand the functions and potentially opposing regulations mediated by HIF-1 and HIF-2. 

According to Bäcker et al. (2017), the lower inflammation during DSS-induced colitis in HIF-1α deficient animals is mainly due to the lower infiltration of immune cells [15]. The increased inflammation during DSS-induced colitis in HIF-2α deficient animals, in turn, may therefore be due to an increased infiltration of immune cells. Although there was no increased expression of *Adgre1* (which encodes for F4/80; Figure 7) we detected increased numbers of F4/80 positive macrophages in the colon tissue of *LysM^Cre/+^ Hif-2α^fl/fl^* and *LysM^Cre/Cre^ Hif-2α^fl/fl^* animals (Figure 5). This discrepancy might be because changes on the mRNA level precede the changes on protein levels and therefore would have been more obvious shortly before the observed time point. In addition, the absolute numbers of macrophages in the whole colon are still relatively low and locally restricted to the inflamed areas. This might cover mRNA changes detected in whole colon tissue. Furthermore, increased infiltration and migration of MPO positive neutrophils into the colon tissue (Figure 6) and an increased gene expression of *Ly6g* in HIF-2α deficient animals during DSS-induced colitis was evident (Figure 7). RNA samples from DSS-treated *LysM^Cre/Cre^ Hif-2α^fl/fl^* animals also tended to show an increased expression of *Cxcl1* (Figure 10), a chemokine attracting neutrophils [44]. However, since we already detected elevated numbers of neutrophils on day 6, we assumed that the expression of *Cxcl1* peaked at an earlier time point. De Filippo et al. (2008) argued that the time of maximum CXCL1 expression in their mouse model of peritonitis peaks earlier than the maximum of neutrophil recruitment [45]. The same group and others have published that mainly proinflammatory macrophages in the gut express CXCL1 and thereby promote the infiltration of neutrophils [46,47]. Lin et al. (2018) also observed an increased infiltration of neutrophils and a higher tissue inflammation after DSS induction in *LysM^Cre/+^ Arnt^fl/fl^* animals [40]. Neutrophils can perform inflammation-promoting functions [47,48]. According to Lin et al. (2018), the ARNT knockout in macrophages led to an increased expression of *Cxcl1* and an increased infiltration of neutrophils, which further promoted inflammation [40]. As the myeloid HIF-2α knockout led to an increased expression of *Cxcl1* in the colon, we assumed that HIF-2α is the predominant HIF-α subunit that normally prevents an overwhelming recruitment of proinflammatory neutrophils under inflammatory conditions. An “isoform switch” might at least in part be responsible for this: if HIF-1α as the proinflammatory HIF isoform might be overactive in the macrophages and neutrophils lacking functional HIF-2α this might drive the inflammation. Support for this theory provides the finding that increased numbers of macrophages and neutrophils came along with an increased expression of proinflammatory cytokines (Figure 10) and that at least some of these cytokines are likely to be direct or indirect HIF-1 target genes: *Il6, Il1b*, *Tnfa*, and *Il10* showed reduced expressions after knockdown of Hif-1a [15,49]. We did not observe an increased hypoxic stabilization of HIF-1α in *LysM^Cre/+^ Hif-2α^fl/fl^* or *LysM^Cre/Cre^ Hif-2α^fl/fl^* animals (Figure S1C) but this might be different in inflamed tissues.

The expression of the proinflammatory cytokine IFNγ is highest in samples from DSS-treated *LysM^Cre/+^ Hif-2α^fl/fl^* animals (Figure 10). The decrease in expression observed in samples from DSS-treated *LysM^Cre/Cre^ Hif-2α^fl/fl^* animals could be due to the abundance of higher concentrations of anti-inflammatory modulators (Tregs; TGFß1, IL10, see below) in these mice or follow a different time course. Mononuclear cells in the intestinal lamina propria of IBD patients showed an increased IFNγ expression [50,51,52] and an IFNγ knockout in mice prevents inflammation of the colon [53]. Thus, the higher *Ifng* expression is likely to promote colitis progression.

*Tnfa* expression was slightly elevated in all DSS-treated animals but showed no clear differences between the genotypes (Figure 10). Macrophages are the main producers of TNFα [54] and a loss of HIF-1α in myeloid cells evoked low *Tnfa* expression in the fatty tissue of a vascular injury model [55]. Bäcker et al. (2017) also observed low *Tnfa* expression in the colon of DSS-treated mice with a HIF-1α deficiency in myeloid cells [15]. However, we could not confirm a HIF-dependency of the *Tnfa* expression in the colon, neither in *LysM Hif-2α^fl/fl^* nor in *LysM^Cre/+^ Hif-1α^fl/fl^* × *Hif-2α^fl/fl^* mice.

The proinflammatory cytokine *Il23* showed a significantly lower expression in samples from DSS-treated *Hif-2α^fl/fl^* and *LysM^Cre/+^ Hif-2α^fl/fl^* animals compared to the control group (Figure 10). IL23 is produced by DCs and macrophages and is involved in the Th17-cell activation and cytokine production [56]. Samples from DSS-treated *LysM^Cre/Cre^ Hif-2α^fl/fl^* animals showed a significantly higher expression of *Il23* compared to samples from the other DSS-treated *Hif-2α^fl/fl^* animals, but the expression was still much lower than in the control groups (Figure 10). IL23 plays an important role in T-cell mediated colitis and promotes the production of *Il17a* [57]. A loss of the IL-23 receptor led to lower IL-17A expression during DSS-induced colitis [58]. Mice with a HIF-1α knockout in DCs or T-cells show a higher *Il23* expression in the colon after DSS treatment [18,38]. However, we observed an upregulation of *Il17a* in samples from DSS-treated animals compared to the control groups (Figure 10). Therefore, it is possible that we missed the *Il23* peak expression, as it has to precede the induction of *Il17a*. Samples from DSS-treated *LysM^Cre/Cre^ Hif-2α^fl/fl^* animals tended to show a higher *Il17a* expression than the samples from the other animals (Figure 9). Lin et al. (2011) observed cells positive for neutrophilic elastase and IL17 in human psoriasis using immunohistochemical staining [59]. Thus, the increased *Il17a* expression in the qPCR data might be partly due to the increased numbers of neutrophils but also due to elevated numbers of T-cells in the colon.

*Cd4* and *Cd8a* gene expression increased in the colon (Figure 7) and expression of *Ccr9* in the lymph nodes of DSS-treated *LysM^Cre/Cre^ Hif-2α^fl/fl^* mice (Figure 9), indicating an increased migration of T-cells towards the gut. CCR9 is expressed by DCs and lymphocytes and promotes the migration of these cells into the gut [32,33]. Higher CCR9 expression might be due to elevated expression of proinflammatory cytokines, especially *Il6* (Figure 10). Choe et al. (2014) have shown that an overexpression of HIF-2α in macrophages suppressed whereas a *Hif-2a* knockdown increased the *Il6* mRNA expression via arginase-1 dependent mechanisms [60]. Despite the lower abundancy of HIF-2α in myeloid cells of *LysM^Cre/+^ Hif-2α^fl/fl^* and *LysM^Cre/Cre^ Hif-2α^fl/fl^* animals we saw an about 2.5-fold increase of the *Arg1* expression in the colon after 6 days of the DSS treatment (Figure 10). This was somewhat unexpected, because not only Choe et al. (2014) but also the group of Takeda et al. (2010) had shown that HIF-2α regulated the *Arg1* expression and thereby determined an anti-inflammatory phenotype of macrophages [60,61]. Potentially, other cells than of the myeloid lineage are responsible for the observed increase in *arg-1* expression in our colon samples or the *arg-1* expression shows a biphasic expression pattern and is induced later by the ongoing inflammation. It is likely that either the loss of HIF-2α indirectly shapes the *Il6* levels found here or that indeed HIF-1α can take over as predominant HIF isoform when HIF-2α is missing and then directly might induce *Il6* that plays a central role in the pathogenesis of IBD. IBD patients showed elevated levels of IL6 in the *lamina propria mucosae* [62]. Together with the soluble IL6 receptor sIL-6R, IL6 forms a complex that can trigger a proinflammatory immune response [63]. sIL6R in turn is expressed by neutrophils [64]. This so-called IL6-trans-signaling process inhibits apoptosis of mucosa-associated T-cells and thereby enables the progression of IBD. Neutralization of this complex in MC patients led to an increased T-cell apoptosis and reduced symptoms [63]. Furthermore, IL6 inhibits the differentiation of naive T-cells into Tregs [65]. DSS-treated *LysM^Cre/+^ Hif-2α^fl/fl^* and *LysM^Cre/Cre^ Hif-2α^fl/fl^* animals show a higher *Foxp3* expression (Figure 7) and high numbers of CD3-FoxP3 double-positive Tregs in the inflamed colon in this study (Figure 8A). The higher expression of *Foxp3* is likely to correlate with higher numbers of regulatory T-cells in the colon that are necessary to control the more pronounced inflammation in these mice. It goes along with an increased *Tgfb1* and *Il10* expression in samples from DSS-treated *Hif-2α^fl/fl^*, *LysM^Cre/+^ Hif-2α^fl/fl^* and *LysM^Cre/Cre^ Hif-2α^fl/fl^* animals (Figure 10). Li et al. (2015) found that the IL6 pretreatment of primary intestinal muscle cells from MC patients increased the TGFβ1 activity and the *Tgfb1* expression in these cells [66]. IL10-deficient mice in turn develop a spontaneous colitis [67]. Thus, the increased *Il10* expression in HIF-2α deficient animals should have a protective effect during colitis. Our finding of higher *Il10* expression in *LysM^Cre/Cre^ Hif-2α^fl/fl^* animals compared to *LysM^Cre/+^ Hif-2α^fl/fl^* mice might explain that the DAI of *LysM^Cre/Cre^ Hif-2α^fl/fl^* animals did not further increase after day 5–6 but remained stable indicating a control of the inflammation (Figure 1B).

Thus, we postulated that a HIF-2α knockout in macrophages favors an increased *Il6* expression that is of central importance for the subsequently more pronounced colon inflammation. Herein with a myeloid HIF-2α knockout, we saw an induction of (i) other proinflammatory cytokines than *Il6* such as *Il23*, *Il17*, and *Ifng* and (ii) a higher recruitment of proinflammatory cells like neutrophils caused by increased *Cxcl1* and T-cells driven by a gradient of *Ccr9*. This is likely to promote colitis progression. The accompanying induction of anti-inflammatory mediators like *Il-10* and *Tgfb* and cells (Tregs) might then limit the progress of colitis.

Mice carrying a double knockout for both HIF-α isoforms in cells of the myeloid lineage exhibited changes neither in the recruitment of immune cells nor in pro- and anti-inflammatory cytokines during DSS colitis. Therefore, we suggested that HIF-1α and HIF-2α fulfill opposing tasks during the activation of myeloid cells and hereby balance each other. This highlights the demand to understand the roles of the HIF isoforms better and urges the need to develop drugs with isoform-specific efficacy.

## 4. Materials and Methods

### 4.1. Animal Model

To investigate the significance of HIF-αs in inflammatory bowel diseases, transgenic mouse strains with a C57BL/6J background were used for animal experiments. The animal experiments were performed with mice showing a conditional knockout of Hif-1a and Hif-2a or a double knockout of Hif-1a and Hif-2a in myeloid cells (*LysM^Cre/+^ Hif-1α^fl/fl^, LysM^Cre/**+**^ Hif-2α^fl/fl^, LysM^Cre/**Cre**^ Hif-2α^fl/fl^,* or *LysM^Cre/+^ Hif-1α^fl/fl^ Hif-2α^fl/fl^*). *Hif-1α^fl/fl^-* (B6.129-*Hif1a^tm3Rsjo^*/J), *Hif-2α^fl/fl^*- (STOCK *Epas1^tm1Mcs^*/J), and LysM-Cre- (B6.129P2-*Lyz2^tm1(cre)Ifo^*/J) animals were acquired via the JAX laboratory. All mice lived under specific pathogen free conditions with a constant night-day-rhythm (12 h/12 h) and received pelleted food and drinking water ad libitum. We analyzed lower numbers of *Hif-1α^fl/fl^* and *LysM^Cre/+^ Hif-1α^fl/fl^* mice because we have already published the effects of an acute colitis on these mice before [15]. The mice we analyzed herein were not identical with the mice published before but served as direct controls for the data gained from the other animals.

The approval of animal experimental work was granted by the Landesamt für Natur, Umwelt und Verbraucherschutz Nordrhein-Westfalen (LANUV NRW) under the reference 84-02.04.2014.A503 at 02/24/2015.

For the induction of colitis, we used mice that were at least 10 weeks old. The mice received drinking water with 2.5% dextran sodium sulfate (MP Biomedicals, Eschwege, Germany; molecular weight: 36–50 kDa) for 6 days. On the 3rd day, the 2.5% DSS water was renewed. Control mice received drinking water without DSS. Body weight, stool consistency, and the presence of blood in the stool were documented daily. These values determined a disease activity index (DAI) [22] to document the severity of the disease (Table 1):

### 4.2. Cell Culture

Bone-marrow derived macrophages (BMDM) were isolated from femurs of mice and cultured according to protocol of Bäcker et al. (2017) [15]. After 7 days the hypoxic stimulation (Baker Ruskinn Invivo_2_, 1% O_2_/5% CO_2_/94% N_2_) was performed for 6 h or 24 h.

Neutrophils from mice were isolated by using Anti-Ly6G MicroBeads UltraPure (Miltenyi Biotec GmbH, Bergisch Gladbach, Germany) from femurs. The cells were cultivated in RPMI medium (Thermo Fisher Scientific, Dreieich, Germany) with 50 ng/mL GM-CSF-conditioned media. For stabilization of HIF, neutrophils were stimulated with 1 mM DMOG (Biomol GmbH, Hamburg, Germany) for 4 h.

### 4.3. Histology

We fixed tissue from the distal colon in 4% paraformaldehyde, drained, and then embedded it in paraffin. Subsequently, 4 µm thick tissue sections were cut on the rotary microtome and the tissue sections were mounted on SuperFrost^®^ Plus slides. The sections were stained with hematoxylin and eosin (H&E). H&E stained tissue was microscopically analyzed and a colon histology score was determined based on the following scoring system as described by Cooper et al. (1993) [22] (Table 2). The maximum of the histology score (achieved by the addition of the score of all three columns) was 10.

The staining with alcian blue visualized the abundance of mucins. The counter staining was performed with eosin. We quantified the alcian blue staining with the help of ImageJ 1.53, NIH Bethesda, Maryland, USA. We split screenshots showing whole colon diameters into the alcian and the eosin channel with the help of the ColorDeconvolution2 Plugin. Afterwards, the *tunica mucosa* was defined as a region of interest in the binaries of the single color channel and we analyzed the numbers of positive pixels in relation to the overall pixels of the *tunica mucosa*. For visualization of macrophages rat anti-F4/80 antibody (Ab) with a goat antirat secondary Ab and for the visualization of neutrophils a goat antimyeloperoxidase antibody with a rabbit antigoat Ab (#sc2774, Santa Cruz Biotechnology, Heidelberg, Germany) were used. For antigen demasking the tissue sections were boiled in 10% retrieval solution (DAKO Agilent Technologies, Waldbronn, Germany) for 20 min. Slides were blocked with 5% normal goat serum in PBS for the F4/80 staining and with 5% normal rabbit serum for the myeloperoxidase staining. The subsequent peroxidase reaction was performed with the ABC- (Vector Laboratories Biozol, Eching, Germany) and the detection with the DAB-Kit (Vectastain; Vector Laboratories Biozol, Eching, Germany). The quantification of neutrophils was also performed with ImageJ 1.53 on four cross sections of the 400× magnification pictures. We marked the krypts (in the *lamina propria mucosae*) and the tops of the krypts (luminal half of *lamina propria mucosae*) by hand and measured the areas. Afterwards, we used the immunohistochemistry (IHC) Toolbox (functions: DAB-H, Color) to process the picture. We used binaries of the DAB color channel (pictures were eroded and watershed image processing was applied) and utilized the “analyze particles”-function (size: 25–2500 pix^2^; circularity: 0–1) for krypts- and tops of the krypts-areas to count MPO-positive cells. Visualization of HIF-1α and HIF-2α (suppl. Figures S2 and S3) was performed with the help of the CSA II Kit (DAKO Agilent Technologies, Waldbronn, Germany, #K149711) according to the manufacturer’s instructions. The staining for CD3 and FoxP3 was performed in analogy to the DAB protocol but without peroxidase reaction. Slides were incubated with Sudan Black (Sigma Aldrich, Hamburg, Germany #199664-25G) according to the manufacturer’s instructions to diminish the background staining and nuclei were counterstained with DAPI (1:10,000; AppliChem, Darmstadt, Germany # A10010010). Table 3 contains the used antibodies.

### 4.4. Real-Time PCR

RNA isolation of the colonic tissue and the lymph nodes was performed using the NucleoSpin^®^ RNA Kit from Macherey-Nagel GmbH. RNA of cell culture probes were isolated by the acid guanidinium thiocyanate/phenol/chloroform extraction method [68]. Subsequently, cDNA synthesis was performed by M-MLV Reverse Transcriptase (Promega Corporation, Walldorf, Germany) according to the manufacturer’s instructions. For real-time PCR amplification GoTaq polymerase reaction mixture (Invitrogen Thermo Fisher Scientific, Dreieich, Germany ) was used. cDNA was amplified by 40 cycles of 95 °C for 15 s and 60 °C for 15 s with gene specific primers (Invitrogen Thermo Fisher Scientific, Dreieich, Germany; see Table 4). Relative changes in mRNA levels were calculated with *Actb* as a reference standard by the ΔΔCt method. All data are given as induction over the respective wildtype control. The controls of the other genotypes thereby did not markedly change with respect to the wildtype control and are not shown in Figure 7, Figure 9, and Figure 10. They can be found in the (supplementary material Figures S4–S6).

### 4.5. Statistical Analyses

Statistical analyses were performed by using the PRISM^®^ software version 8.0.2 from GraphPad Software Inc, San Diego, USA. Two-way ANOVA analyses were chosen for the statistical evaluation of the data sets with two variables. Data sets of the histological data were tested for normality with the Shapiro–Wilk test and a two-way unpaired parametric *t*-test was performed on these data sets. Data sets of the qPCRs were subjected to a ROUT (robust regression and outlier removal) test [69] before statistical evaluation, so that outliers were excluded from further evaluation. A *p*-value ≤ 0.05 (*p* ≤ 0.05 = *, *p* ≤ 0.01 = **, *p* ≤ 0.001 = ***) was considered statistically significant. All figures indicate means with standard error (SEM).

## Figures and Tables

**Table 1:** Disease activity index.

Score	Weight Loss	Stool Consistency	Hema Screen Test (Occult Blood)
0	none	normal	negative
1	1–5%	normal	negative
2	5–10%	loose	positive
3	10–20%	loose	positive
4	>20%	diarrhea	visible bleeding

**Table 2:** Histology scoring system.

Score	Infiltrating Immune Cells	Extent of Injury	Crypt Damage
0	rare	none	intact crypts
1	slightly dispersed	mucosal	basal 1/3 damaged
2	moderately increased	mucosal and submucosal	basal 2/3 damaged
3	severely large areas: loss of tissue structure	transmural	only surface epithelium intact
4	-	-	loss of entire crypt and epithelium

**Table 3:** Antibodies in immunohistochemistry staining.

Antibody	Concentration	Catalog Number and Company
rat anti-F4/80	0.180556	#MCA497B, Bio-Rad AbD Serotec GmbH, Puchheim, Germany
goat antirat secondary Ab	0.388889	#A11077, Invitrogen Thermo Fisher Scientific, Dreieich, Germany
goat antimyeloperoxidase	0.180556	# AF3667, R&D systems
rabbit antigoat secondary Ab	0.388889	# sc2774, Santa Cruz Biotechnology, Heidelberg, Germany
goat anti-CD3-ε	0.215278	#sc-1127, Santa Cruz Biotechnology, Heidelberg, Germany
rabbit antigoat Alexa Fluor 488	0.388889	#A-11078, Thermo Fisher Scientific, Dreieich, Germany
Rat anti-FoxP3	0.111111	# FJK-16s, eBioscience Thermo Fisher Scientific, Dreieich, Germany
goat antirat Alexa Fluor 568	0.388889	#A-11077, Thermo Fisher Scientific, Dreieich, Germany
rabbit anti-HIF-1alpha (C-Term)	1:10,000	#CAY-10006421, Cayman Chemical Biomol GmbH, Hamburg, Germany
rabbit anti-HIF-2 alpha/EPAS1	1:10,000	#NB100-122, Novus Biologicals, Wiesbaden, Germany
Polyclonal goat-antirabbit Immunoglobulin/HRP	0.388889	#P044801-2, DAKO Agilent Technologies, Waldbronn, Germany

**Table 4:** Primer sequence and product length.

Target Gene	Accession-Number	Sequence	Product Length (bp)
*5′ Adgre1* *3′ Adgre1*	NM_010130	TCTGGGGAGCTTACGATGGA GAATCCCGCAATGATGGCAC	237
*5′ Arg1* *3′ Arg1*	NM_007482	AACACGGCAGTGGCTTTAACC GGTTTTCATGTGGCGCATTC	117
*5′ Ccr9* *3′ Ccr9*	NM_001166625	CCAAGGTGCCCACAATGAAC ACTCACAAGCCTTATTCCTGGC	179
*5′ Cd4* *3′ Cd4*	NM_013488	TGAAGGAAACGCTCCCACTC AGCAGTGCTGATGTCTTGCT	136
*5′ Cd8a* *3′ Cd8a*	NM_001081110	ACCCTTGGCCGGAATCTGCG CTGTCTGACTAGCGGCCTGGGA	112
*5′ Cd11c* *3′ Cd11c*	NM_021334	GGACGGTGCTGAGTTCGGACACAG CCACAAGCCAACAGCCAGGAAGG	231
*5′ Cxcl1* *3′ Cxcl1*	NM_008176	CAGGGTCAAGGCAAGCCTC CTGGGATTCACCTCAAGAACATC	117
*5′ Foxp3* *3′ Foxp3*	NM_001199347	CTGGCGAAGGGCTCGGTAGTCCT CTCCCAGAGCCCATGGCAGAAGT	250
*5′Hif-1a exon 2* *3′ Hif-1a exon 2*	NM_001313919	CATCCAGAAGTTTTCTCACACG GGCGAAGCAAAGAGTCTGAA	138
*5′Hif-2a exon 2* *3′ Hif-2a exon 2*	NM_010137	AGGAGACGGAGGTCTTCTATGA ACAGGAGCTTATGTGTCCGA	126
*5′ Ifng* *3′ Ifng*	NM_008337	GGTCAACAACCCACAGGTCC CAGCGACTCCTTTTCCGCTT	105
*5′ Il6* *3′ Il6*	NM_031168	TCCTACCCCAATTTCCAATGC CATAACGCACTAGGTTTGCCG	151
*5′ Il10* *3′ Il10*	NM_010548	TGCCCCAGGCAGAGAAGCAT GGGAGAAATCGATGACAGCGCC	109
*5′ Il23a* *3′ Il23a*	NM_031252	ACCAGCGGGACATATGAATCT AGACCTTGGCGGATCCTTTG	147
*5′ Il17a* *3′ Il17a*	NM_010552	TCATCCCTCAAAGCTCAGCG TTCATTGCGGTGGAGAGTCC	167
*5′ Ly6g* *3′ Ly6g*	NM_023463	GTACCTTGGGAAGATGTGGGT GTTCAGGCCCAGCTTATGGT	103
*5′ Tgfb1* *3′ Tgfb1*	NM_011577	TGGCCAGATCCTGTCCAAAC CATAGATGGCGTTGTTGCGG	215
*5′ Tnfa* *3′ Tnfa*	NM_011609	TACCTCCTCCGCTTGCAAAT GAGTAGACTTCGGGCCTCCAC	151
*5′ Actb* *3′ Actb*	NM_007393	TAGGCACCAGGGTGTGATGG CTCGGTGAGCAGCACAGG	208

## Supplementary Materials

Supplementary materials can be found at https://www.mdpi.com/1422-0067/21/22/8551/s1. Figure S1: Knockout efficiency in bone marrow-derived macrophages (BMDM) and neutrophils; Figure S2: Expression of HIF-1α is restricted to the epithelial tips in control mice but spreads towards deeper layers with increasing inflammation; Figure S3: Expression of HIF-2α is restricted to the epithelial tips in control mice but spreads towards deeper layers with increasing inflammation; Figure S4: Gene expression of immune cell markers of control animals; Figure S5: Gene expression of *Ccr9* in lymph nodes of control animals; Figure S6: Gene expression of pro- and antiinflammatory cytokines of control animals; Table S1: Gene expression of immune cell markers of control animals; Table S2: Gene expression of Ccr9 in lymph nodes of control animals; Table S3: Gene expression of pro- and anti-inflammatory cytokines of control animals.

## Abbreviations

BMDM	Bone-marrow-derived macrophage
CCR9	C-C chemokine receptor type 9
C-TAD	C-terminal transactivation domain
CXCL1	chemokine (C-X-C motif) ligand 1
DAI	*Disease Activity Index*
DC	Dendritic cell
DNA	deoxyribonucleic acid
DSS	*dextran* sodium sulfate
FIH	*Factor Inhibiting HIF*
HIF	Hypoxia-inducible factor
HRE	Hypoxia-Response Element
IBD	Inflammatory bowel disease
IFNγ	Interferon-γ
IL	Interleukin
ODD	Oxygen-dependent degradation
O_2_	Oxygen
PHD	Prolyl hydroxylase
pVHL	von-Hippel Lindau protein
TGFβ	Transforming growth factor beta
TNFα	Tumor necrosis factor alpha
