# Supplementary material for: The Importance of Hypoxia-Inducible Factors (HIF-1 and HIF-2) for the Pathophysiology of Inflammatory Bowel Disease
Source: Int J Mol Sci. 2020 Nov 13;21(22):8551. doi: 10.3390/ijms21228551 (PMC7697655; doi:10.3390/ijms21228551)
Supplement: Supplementary file 1 [file ijms-21-08551-s001.pdf]

International Journal of *Molecular Sciences*

MDPI

Supplementary Materials

BMDMs

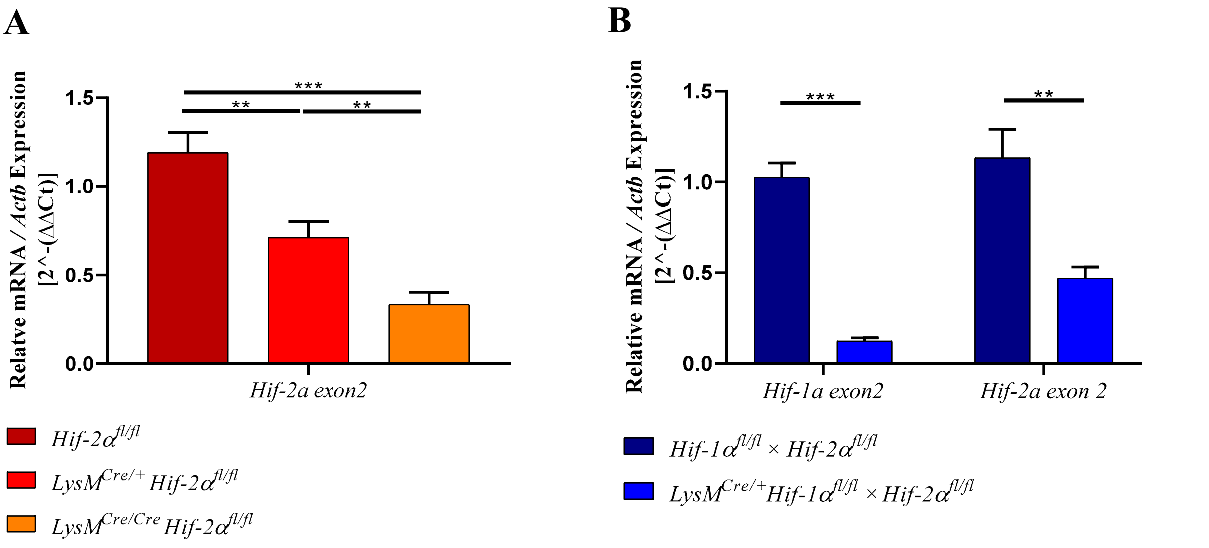

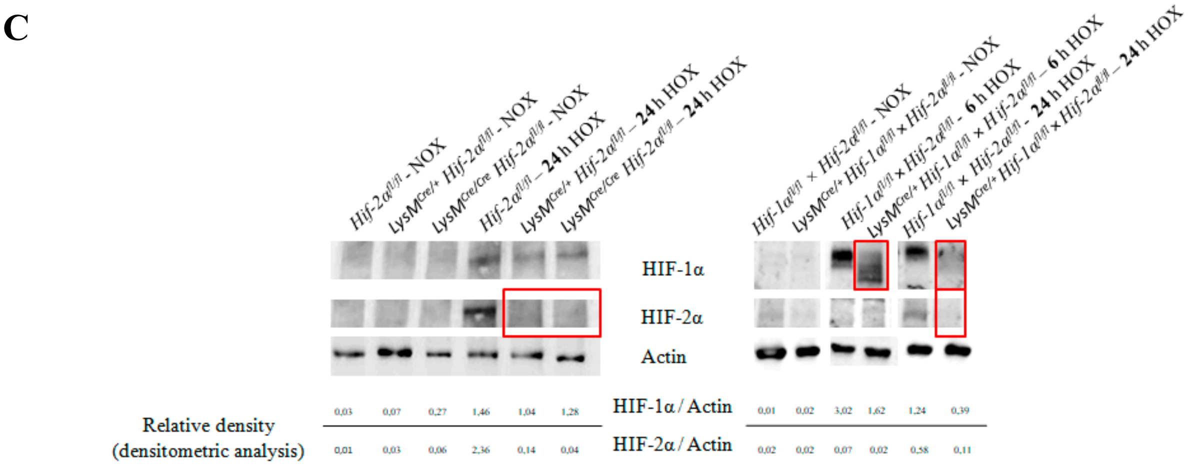

Neutrophils

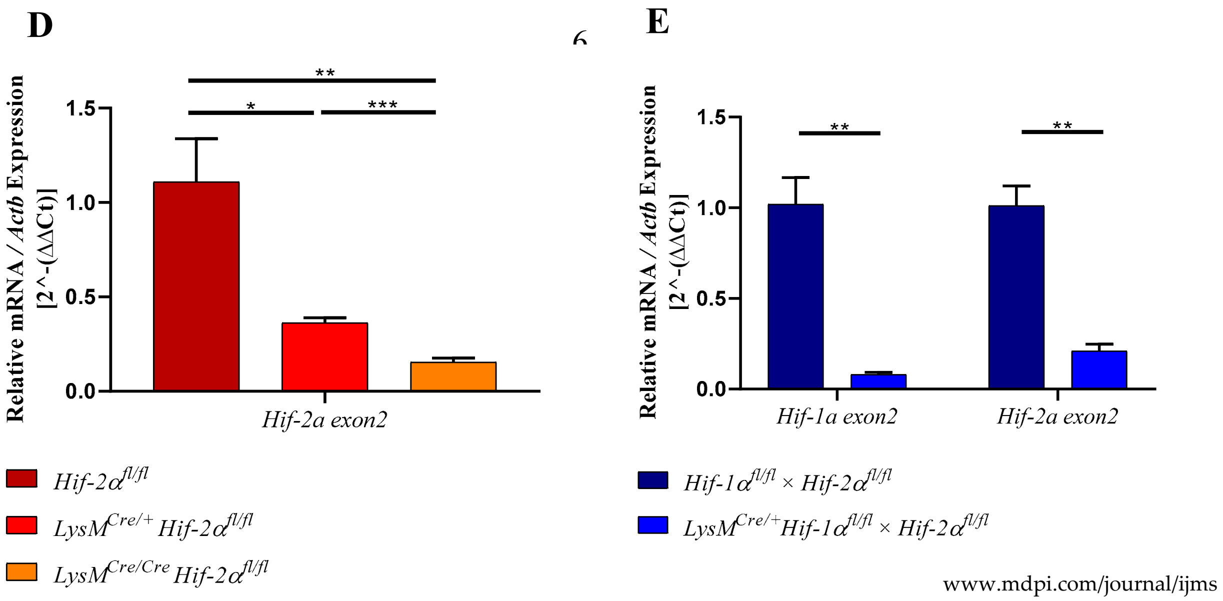

Figure S1: Knockout efficiency in bone marrow-derived macrophages (BMDM) and neutrophils. For the detection of the knockout efficiency BMDMs were cultivated for 6 h or 24 h under hypoxic conditions (HOX, 1 % O_2) and mRNA and protein were isolated. Knockout efficiency was determined by qPCR with primers specific for *Hif2a exon 2* (after 24 h HOX) or *Hif1a exon 2* (after 6 h HOX) (A, B). For protein analysis the BMDM treatment is indicated in (C; HOX = 1% O_2). Whole cell protein lysates (50 µg per lane) have been subjected to immunoblot analysis with specific antibodies against HIF-1α and HIF-2α. Cre abundance induced a shorter protein of HIF-1α that is still detectable by the antibody (upper right part of (C)). The densitometric analysis of HIF-α bands in correlation with actin bands is given beneath each lane and revealed a lower expression of HIF-2α in both knockout strains and an additionally lower abundancy of the longer form of HIF-1α in BMDMs of $LysM^{Cre/+}$ $Hif\text{-}1\alpha^{fl/fl}$ × $Hif\text{-}2\alpha^{fl/fl}$ mice. To detect knockout efficiency in neutrophils, mRNA was isolated after cell cultivation for 4 h with 1 mM DMOG (dimethyloxalylglycine). The knockout efficiency was determined in accordance to BMDMs (D,E). Statistical analysis was performed with an unpaired t-test (mean values ± SEM; n= 3 / 4). *: $p < 0.05$; **: $p < 0.01$; ***: $p < 0.001$.

HIF1

A

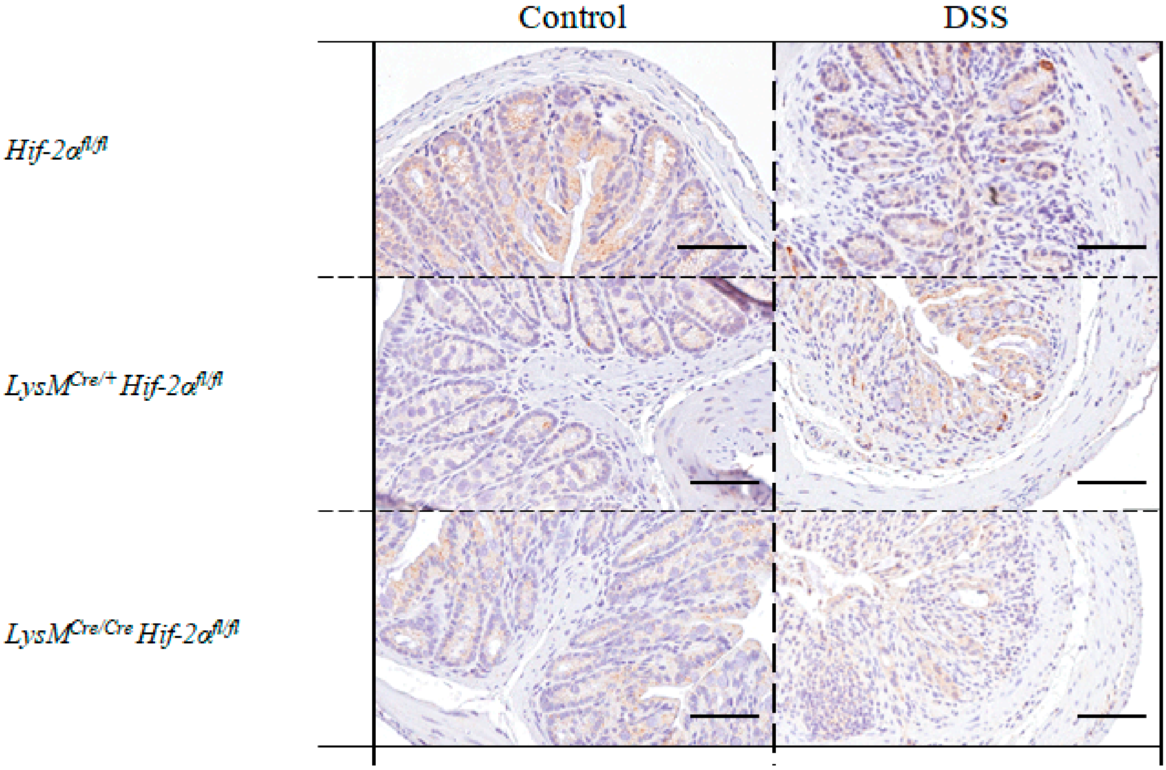

B

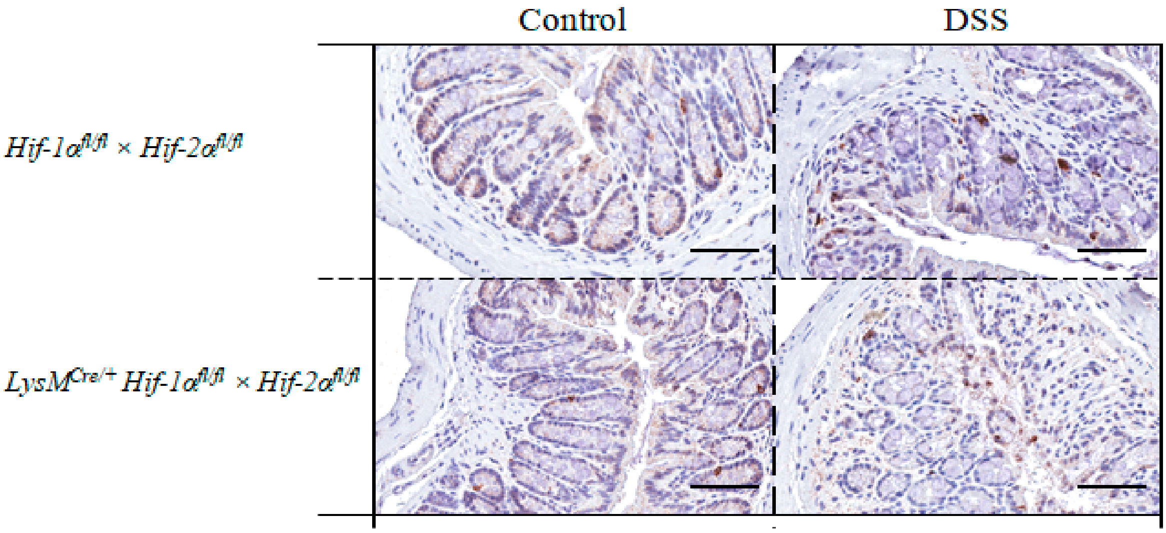

Figure S2: Expression of HIF-1*α* is restricted to the epithelial tips in Control mice but spreads towards deeper layers with increasing inflammation. Exemplary presentation of HIF-1*α*-stained colon tissue sections of (A) *Hif-2α*$^{fl/fl}$, *LysM*$^{Cre/+}$ *Hif-2α*$^{fl/fl}$ and *LysM*$^{Cre/Cre}$ *Hif-2α*$^{fl/fl}$ animals, and (B) *Hif-1α*$^{fl/fl}$ × *Hif-2α*$^{fl/fl}$ and *LysM*$^{Cre/+}$ *Hif-1α*$^{fl/fl}$ × *Hif-2α*$^{fl/fl}$ animals with and without DSS treatment. After DSS treatment, HIF-1*α* expression is no longer limited to the epithelial tips but can also be found in invaded immune cells in the *lamina propria mucosae* and *tela submucosa*. (A): *n*(Control)= 3 (*LysM*$^{Cre/Cre}$ *Hif-2α*$^{fl/fl}$) / 5 (*Hif-2α*$^{fl/fl}$, *LysM*$^{Cre/+}$ *Hif-2α*$^{fl/fl}$), *n*(DSS)= 8 (*LysM*$^{Cre/Cre}$ *Hif-2α*$^{fl/fl}$) / 12 (*Hif-2α*$^{fl/fl}$, *LysM*$^{Cre/+}$ *Hif-2α*$^{fl/fl}$); (B): *n*(Control)=7, *n*(DSS)= 14. (Magnification 200× and scale bar: 100 μm).

HIF2

A

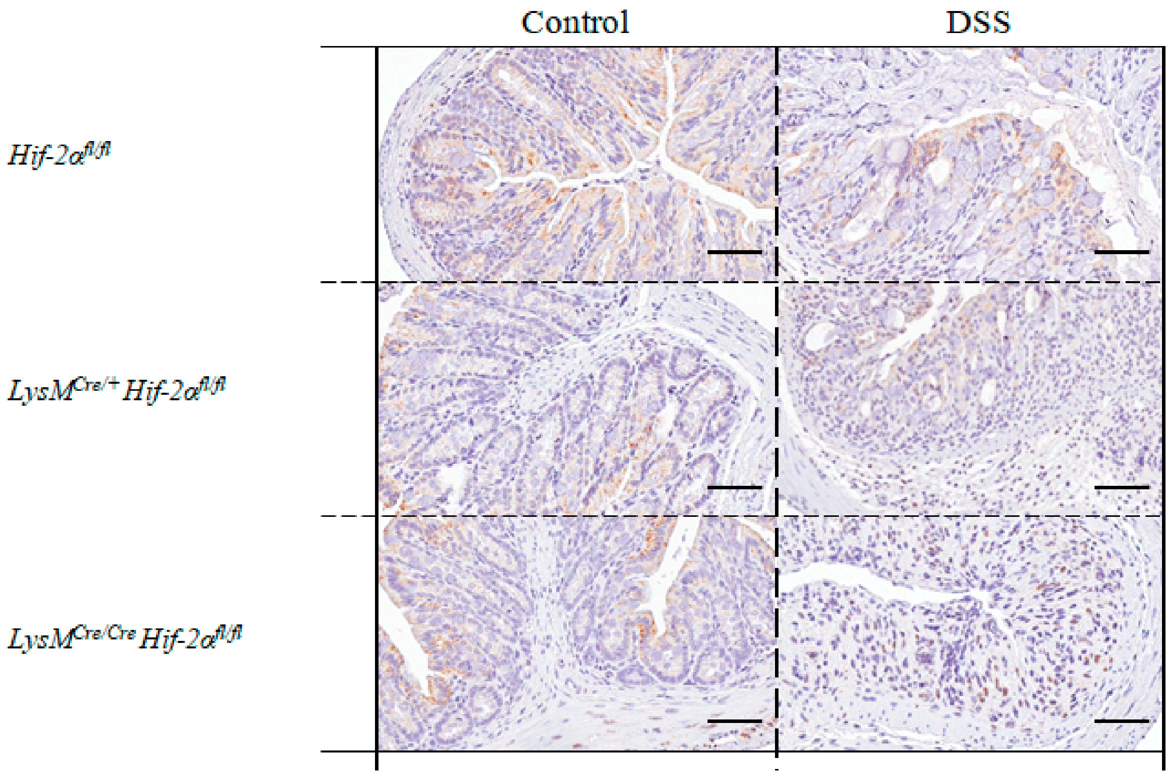

B

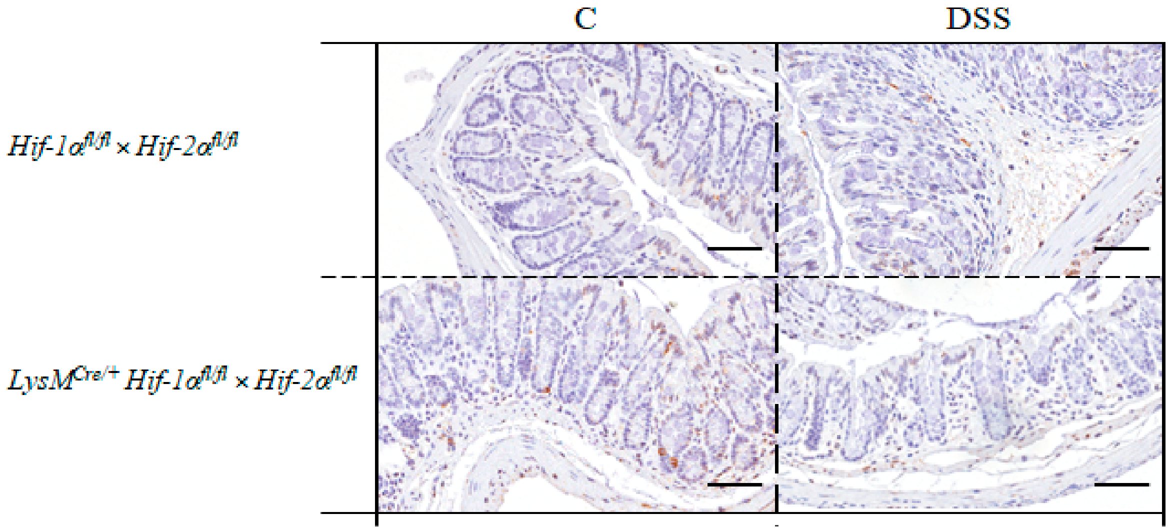

Figure S3: Expression of HIF-2α is restricted to the epithelial tips in Control mice but spreads towards deeper layers with increasing inflammation. Exemplary presentation of HIF-2α-stained colon tissue sections of (A) *Hif-2α*$^{fl/fl}$, *LysM*$^{Cre/+}$ *Hif-2α*$^{fl/fl}$ and *LysM*$^{Cre/Cre}$ *Hif-2α*$^{fl/fl}$ animals, and (B) *Hif-1α*$^{fl/fl}$ × *Hif-2α*$^{fl/fl}$ and *LysM*$^{Cre/+}$ *Hif-1α*$^{fl/fl}$ × *Hif-2α*$^{fl/fl}$ animals with and without DSS treatment. After DSS treatment, HIF-2α expression is no longer limited to the epithelial tips but can also be found in invaded immune cells in the *lamina propria mucosae* and *tela submucosa*. (A): *n*(Control)= 3 (*LysM*$^{Cre/Cre}$ *Hif-2α*$^{fl/fl}$) / 5 (*Hif-2α*$^{fl/fl}$, *LysM*$^{Cre/+}$ *Hif-2α*$^{fl/fl}$), *n*(DSS)= 8 (*LysM*$^{Cre/Cre}$ *Hif-2α*$^{fl/fl}$) / 12 (*Hif-2α*$^{fl/fl}$, *LysM*$^{Cre/+}$ *Hif-2α*$^{fl/fl}$); (B): *n*(Control)=7, *n*(DSS)= 14. (Magnification 200× and scale bar: 100 μm).

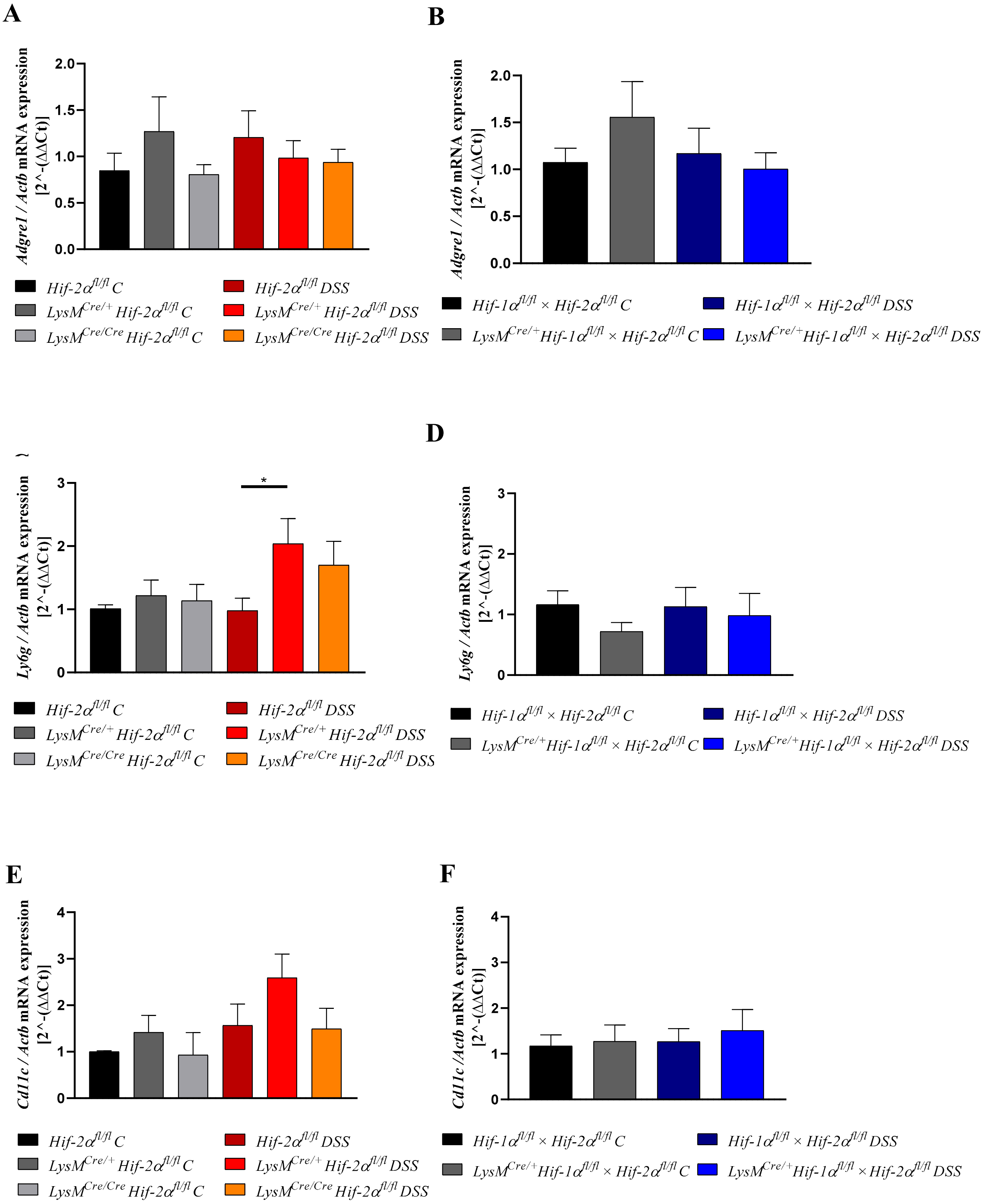
A
B
C
D
E
F
Adgre1 / Actb mRNA expression [2^-(ΔΔCt)]
Ly6g / Actb mRNA expression [2^-(ΔΔCt)]
Cd11c / Actb mRNA expression [2^-(ΔΔCt)]
0.0
0.5
1.0
1.5
2.0
0
1
2
3
4
*
Hif-2α$^{fl/fl}$ C
LysM$^{Cre/+}$ Hif-2α$^{fl/fl}$ C
LysM$^{Cre/Cre}$ Hif-2α$^{fl/fl}$ C
Hif-2α$^{fl/fl}$ DSS
LysM$^{Cre/+}$ Hif-2α$^{fl/fl}$ DSS
LysM$^{Cre/Cre}$ Hif-2α$^{fl/fl}$ DSS
Hif-1α$^{fl/fl}$ × Hif-2α$^{fl/fl}$ C
LysM$^{Cre/+}$Hif-1α$^{fl/fl}$ × Hif-2α$^{fl/fl}$ C
Hif-1α$^{fl/fl}$ × Hif-2α$^{fl/fl}$ DSS
LysM$^{Cre/+}$Hif-1α$^{fl/fl}$ × Hif-2α$^{fl/fl}$ DSS

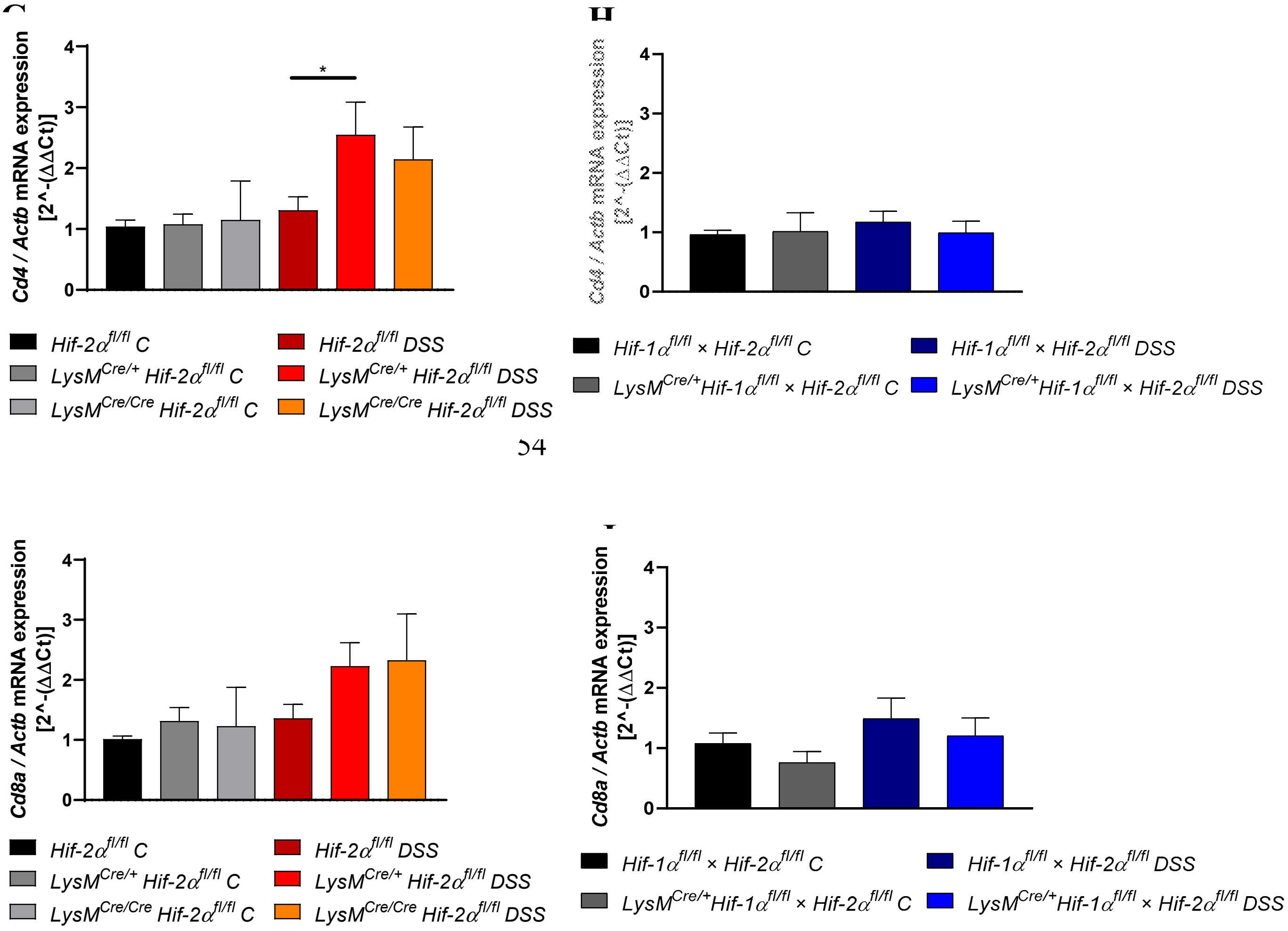

K

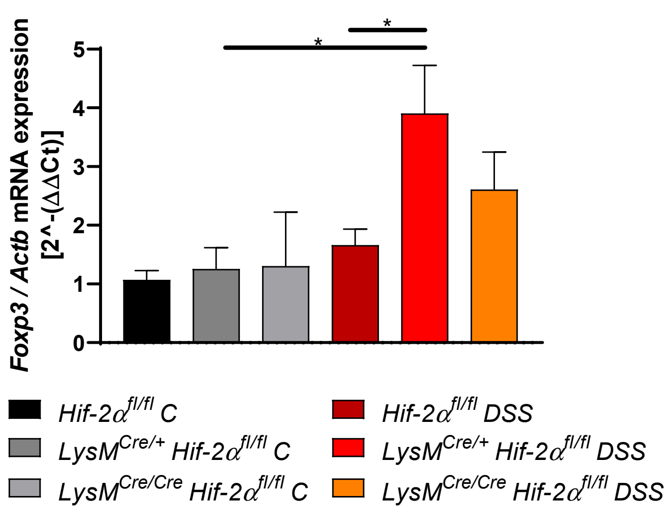

Figure S4: Gene expression of immune cell markers of control animals. Quantitative determination of the gene expression in the colon tissue of the control groups by qPCR. Statistical analysis was performed with an unpaired t-test (mean values ± SEM; n(C)= 3 (*LysM*$^{Cre/Cre}$ *Hif-2α*$^{fl/fl}$) / 6/7 (*Hif-2α*$^{fl/fl}$; *LysM*$^{Cre/+}$ *Hif-2α*$^{fl/fl}$, *Hif-1α*$^{fl/fl}$ × *Hif-2α*$^{fl/fl}$, *LysM*$^{Cre/+}$ *Hif-1α*$^{fl/fl}$ × *Hif-2α*$^{fl/fl}$), n(DSS)= 6 (*LysM*$^{Cre/Cre}$ *Hif-2α*$^{fl/fl}$) / 14/15 (*Hif-2α*$^{fl/fl}$; *LysM*$^{Cre/+}$ *Hif-2α*$^{fl/fl}$, *Hif-1α*$^{fl/fl}$ × *Hif-2α*$^{fl/fl}$, *LysM*$^{Cre/+}$ *Hif-1α*$^{fl/fl}$ × *Hif-2α*$^{fl/fl}$)). *: $p < 0.05$.

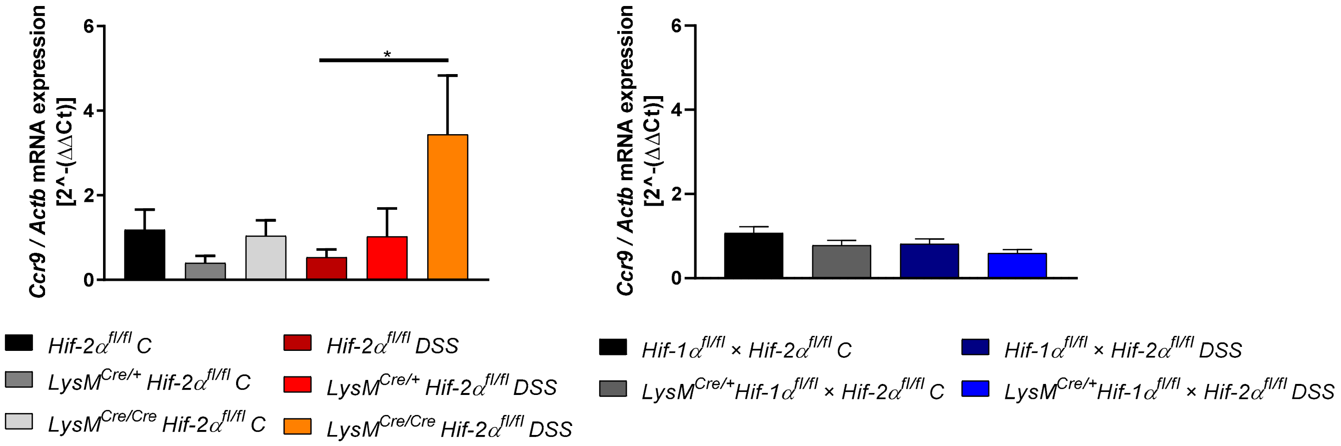

Figure S5: Gene expression of *Ccr9* in lymph nodes of control animals. Quantitative determination of the *Ccr9* expression in the colon tissue of the control groups by qPCR. Statistical analysis was performed with an unpaired t-test (mean values ± SEM**;** *n*(C)= 3 (*LysM*$^{Cre/Cre}$ *Hif-2α*$^{fl/fl}$) / 6/7 (*Hif-2α*$^{fl/fl}$; *LysM*$^{Cre/+}$ *Hif-2α*$^{fl/fl}$, *Hif-1α*$^{fl/fl}$ × *Hif-2α*$^{fl/fl}$, *LysM*$^{Cre/+}$ *Hif-1α*$^{fl/fl}$ × *Hif-2α*$^{fl/fl}$), *n*(DSS)= 6 (*LysM*$^{Cre/Cre}$ *Hif-2α*$^{fl/fl}$) / 14/15 (*Hif-2α*$^{fl/fl}$; *LysM*$^{Cre/+}$ *Hif-2α*$^{fl/fl}$, *Hif-1α*$^{fl/fl}$ × *Hif-2α*$^{fl/fl}$, *LysM*$^{Cre/+}$ *Hif-1α*$^{fl/fl}$ × *Hif-2α*$^{fl/fl}$)). *: $p < 0.05$.

A **B**

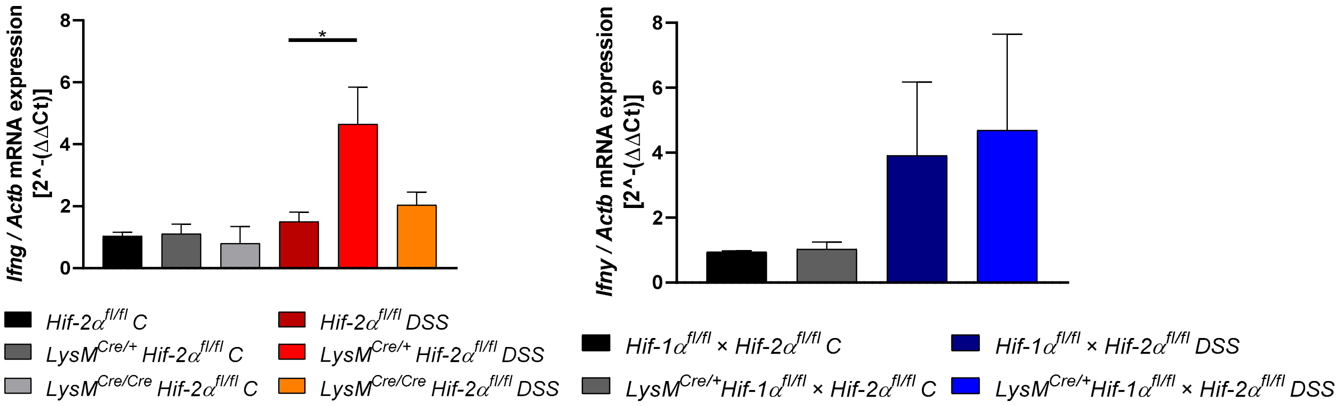

C

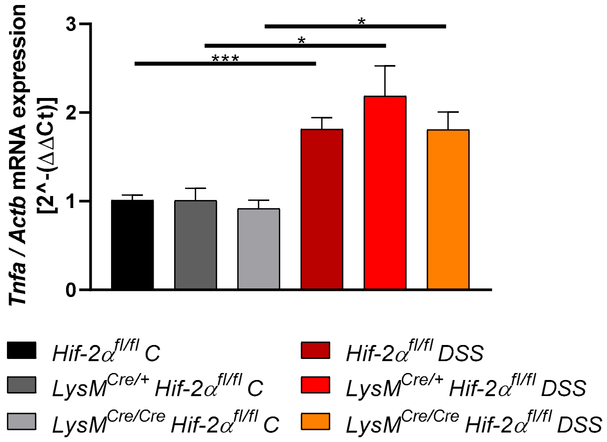
Tnfa / Actb mRNA expression [2^-(ΔΔCt)]
0
1
2
3
***
*
*
Hif-2α fl/fl C
LysM Cre/+ Hif-2α fl/fl C
LysM Cre/Cre Hif-2α fl/fl C
Hif-2α fl/fl DSS
LysM Cre/+ Hif-2α fl/fl DSS
LysM Cre/Cre Hif-2α fl/fl DSS

D

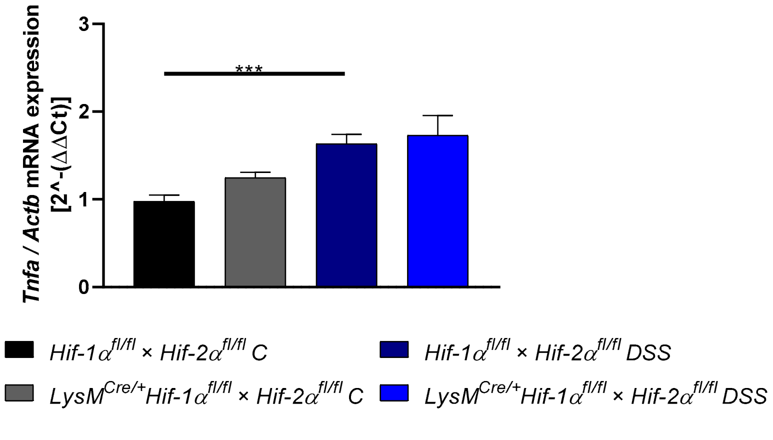
Tnfa / Actb mRNA expression [2^-(ΔΔCt)]
0
1
2
3
***
Hif-1α fl/fl × Hif-2α fl/fl C
LysM Cre/+ Hif-1α fl/fl × Hif-2α fl/fl C
Hif-1α fl/fl × Hif-2α fl/fl DSS
LysM Cre/+ Hif-1α fl/fl × Hif-2α fl/fl DSS

E

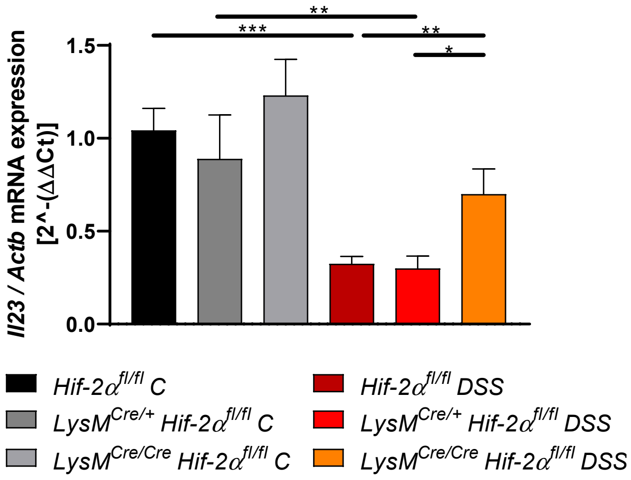
Il23 / Actb mRNA expression [2^-(ΔΔCt)]
0.0
0.5
1.0
1.5
**
***
**
*
Hif-2α fl/fl C
LysM Cre/+ Hif-2α fl/fl C
LysM Cre/Cre Hif-2α fl/fl C
Hif-2α fl/fl DSS
LysM Cre/+ Hif-2α fl/fl DSS
LysM Cre/Cre Hif-2α fl/fl DSS

F

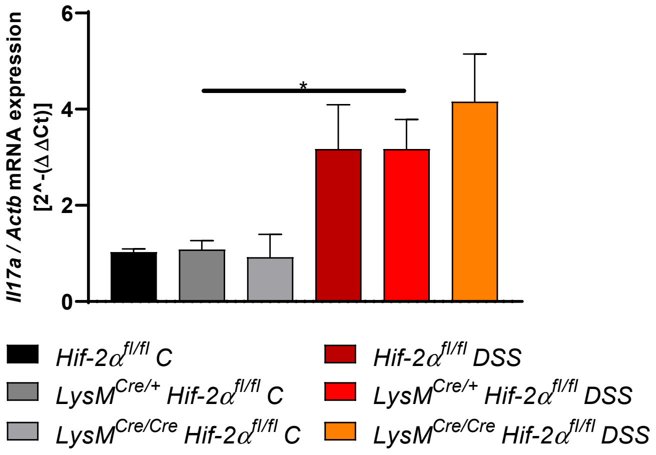
Il17a / Actb mRNA expression [2^-(ΔΔCt)]
0
2
4
6
*
Hif-2α fl/fl C
LysM Cre/+ Hif-2α fl/fl C
LysM Cre/Cre Hif-2α fl/fl C
Hif-2α fl/fl DSS
LysM Cre/+ Hif-2α fl/fl DSS
LysM Cre/Cre Hif-2α fl/fl DSS

G

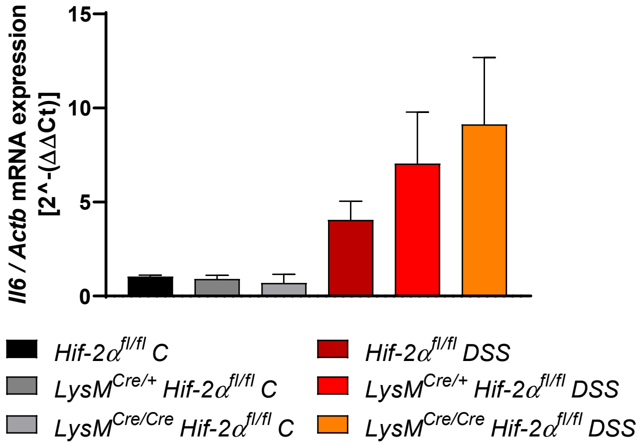
Il6 / Actb mRNA expression [2^-(ΔΔCt)]
0
5
10
15
Hif-2α fl/fl C
LysM Cre/+ Hif-2α fl/fl C
LysM Cre/Cre Hif-2α fl/fl C
Hif-2α fl/fl DSS
LysM Cre/+ Hif-2α fl/fl DSS
LysM Cre/Cre Hif-2α fl/fl DSS

H

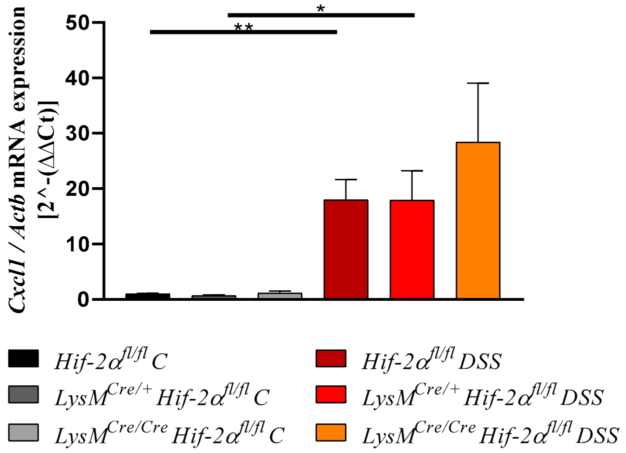
Cxcl1 / Actb mRNA expression [2^-(ΔΔCt)]
0
10
20
30
40
50
*
**
Hif-2α fl/fl C
LysM Cre/+ Hif-2α fl/fl C
LysM Cre/Cre Hif-2α fl/fl C
Hif-2α fl/fl DSS
LysM Cre/+ Hif-2α fl/fl DSS
LysM Cre/Cre Hif-2α fl/fl DSS

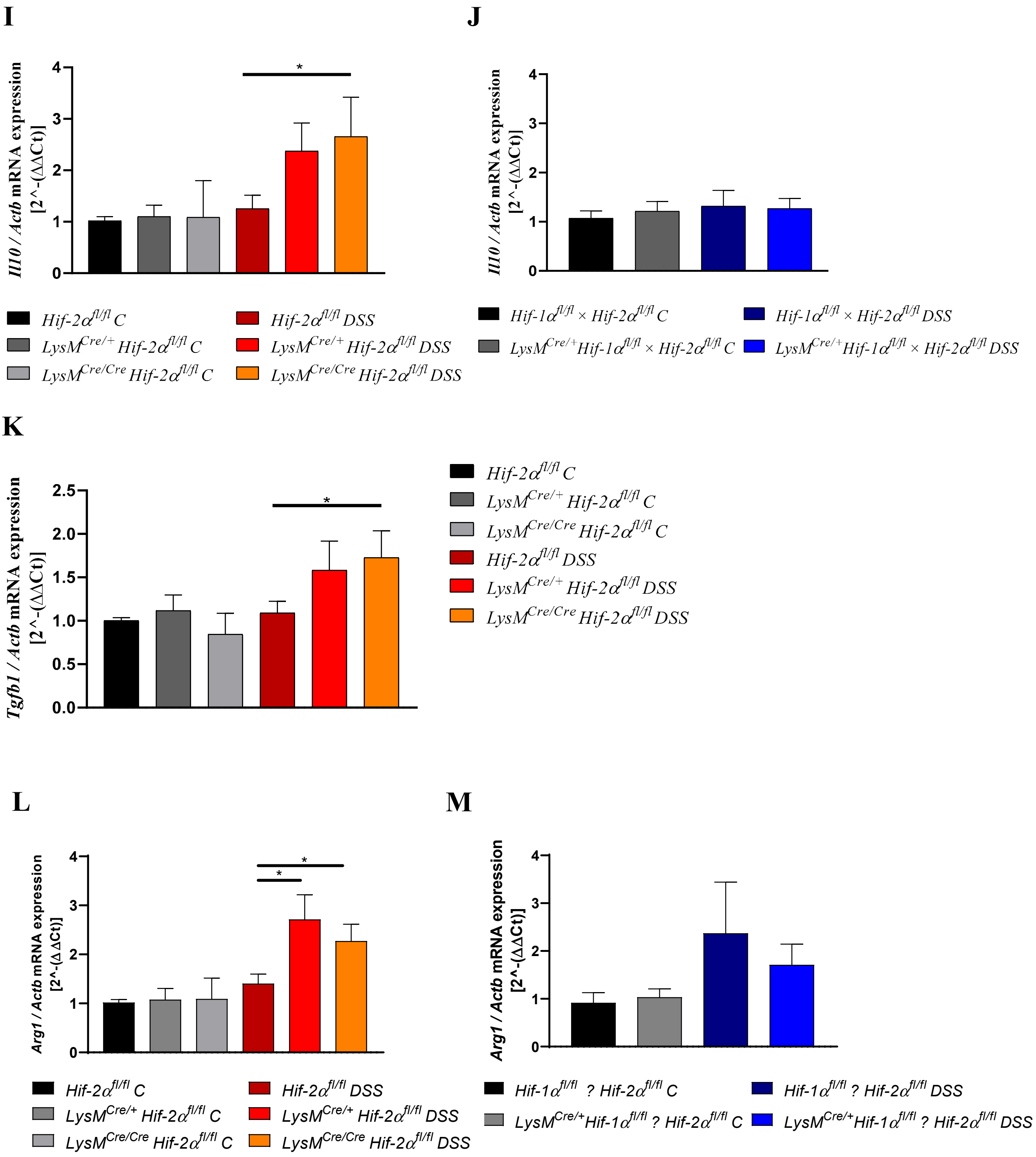

Figure S6: Gene expression of pro- and anti-inflammatory cytokines of control animals. Quantitative determination of the gene expression in the colon tissue of the control groups by qPCR. Statistical analysis was performed with an unpaired t-test (mean values ± SEM; *n*(C)= 3 (*LysM$^{Cre/Cre}$ Hif-2α$^{fl/fl}$*) / 6/7 (*Hif-2α$^{fl/fl}$*; *LysM$^{Cre/+}$ Hif-2α$^{fl/fl}$*, *Hif-1α$^{fl/fl}$* × *Hif-2α$^{fl/fl}$*, *LysM$^{Cre/+}$ Hif-1α$^{fl/fl}$* × *Hif-2α$^{fl/fl}$*), *n*(DSS)= 6 (*LysM$^{Cre/Cre}$ Hif-2α$^{fl/fl}$*) / 14/15 (*Hif-2α$^{fl/fl}$*; *LysM$^{Cre/+}$ Hif-2α$^{fl/fl}$*, *Hif-1α$^{fl/fl}$* × *Hif-2α$^{fl/fl}$*, *LysM$^{Cre/+}$ Hif-1α$^{fl/fl}$* × *Hif-2α$^{fl/fl}$*)). *: $p < 0.05$; **: $p < 0.01$; ***: $p < 0.001$.

Table S1: Gene expression of immune cell markers of control animals. Quantitative determination of the gene expression in the colon tissue of the control groups by qPCR (mean values ± SEM).

Target gene	Animal strain	Mean value $2^{-(\Delta\Delta Ct)}$	± SEM	Number of animals (n)
Adgre1	*Hif-2α^{fl/fl}*<br>*LysM^{Cre/+} Hif-2α^{fl/fl}*<br>*LysM^{Cre/Cre} Hif-2α^{fl/fl}*	0,85<br>1,27<br>0,81	0,187<br>0,373<br>0,105	7<br>6<br>3
	Hif-1α^{fl/fl} × *Hif-2α^{fl/fl}*<br>*LysM^{Cre/+} Hif-1α^{fl/fl}* × *Hif-2α^{fl/fl}*	1,08<br>1,56	0,15<br>0,38	7<br>7
Ly6g	*Hif-2α^{fl/fl}*<br>*LysM^{Cre/+} Hif-2α^{fl/fl}*<br>*LysM^{Cre/Cre} Hif-2α^{fl/fl}*	1,01<br>1,22<br>1,14	0,06<br>0,25<br>0,26	7<br>6<br>3
	Hif-1α^{fl/fl} × *Hif-2α^{fl/fl}*<br>*LysM^{Cre/+} Hif-1α^{fl/fl}* × *Hif-2α^{fl/fl}*	1,16<br>0,72	0,23<br>0,15	7<br>7
Cd11c	*Hif-2α^{fl/fl}*<br>*LysM^{Cre/+} Hif-2α^{fl/fl}*<br>*LysM^{Cre/Cre} Hif-2α^{fl/fl}*	1,00<br>1,42<br>0,93	0,02<br>0,36<br>0,48	7<br>6<br>3
	Hif-1α^{fl/fl} × *Hif-2α^{fl/fl}*<br>*LysM^{Cre/+} Hif-1α^{fl/fl}* × *Hif-2α^{fl/fl}*	1,17<br>1.27	0,24<br>0,36	7<br>7
Cd4	*Hif-2α^{fl/fl}*<br>*LysM^{Cre/+} Hif-2α^{fl/fl}*<br>*LysM^{Cre/Cre} Hif-2α^{fl/fl}*	1,04<br>1,07<br>1,14	0,11<br>0,17<br>0,64	7<br>6<br>3
	Hif-1α^{fl/fl} × *Hif-2α^{fl/fl}*<br>*LysM^{Cre/+} Hif-1α^{fl/fl}* × *Hif-2α^{fl/fl}*	0,96<br>1,02	0,07<br>0,31	7<br>7
Cd8a	*Hif-2α^{fl/fl}*<br>*LysM^{Cre/+} Hif-2α^{fl/fl}*<br>*LysM^{Cre/Cre} Hif-2α^{fl/fl}*	1,01<br>1,31<br>1,22	0,06<br>0,23<br>0,65	7<br>6<br>3

	Hif-1α$^{fl/fl}$ × *Hif-2α$^{fl/fl}$*<br>*LysM$^{Cre/+}$ Hif-1α$^{fl/fl}$* × *Hif-2α$^{fl/fl}$*	1,08<br>0,76	0,17<br>0,18	7<br>7
Foxp3	*Hif-2α$^{fl/fl}$*<br>*LysM$^{Cre/+}$ Hif-2α$^{fl/fl}$*<br>*LysM$^{Cre/Cre}$ Hif-2α$^{fl/fl}$*	1,06<br>1,25<br>1,30	0,16<br>0,37<br>0,93	7<br>6<br>3

Table S2: Gene expression of *Ccr9* in lymph nodes of control animals.

Quantitative determination of the *Ccr9* expression in the colon tissue of the control groups by qPCR (mean values ± SEM).

Target gene	Animal strain	Mean value $2^{-(\Delta\Delta Ct)}$	± SEM	Number of animals (n)
Ccr9	*Hif-2α$^{fl/fl}$*<br>*LysM$^{Cre/+}$ Hif-2α$^{fl/fl}$*<br>*LysM$^{Cre/Cre}$ Hif-2α$^{fl/fl}$*	1,185<br>0,397<br>1,038	0,48<br>0,17<br>0,37	3<br>4<br>3
	Hif-1α$^{fl/fl}$ × *Hif-2α$^{fl/fl}$*<br>*LysM$^{Cre/+}$ Hif-1α$^{fl/fl}$* × *Hif-2α$^{fl/fl}$*	1,03<br>0,92	0,12<br>0,11	6<br>5

Table S 3: Gene expression of pro- and anti-inflammatory cytokines of control animals.

Quantitative determination of the gene expression in the colon tissue of the control groups by qPCR (mean values ± SEM).

Target gene	Animal strain	Mean value $2^{-(\Delta\Delta Ct)}$	± SEM	Number of animals (n)
Ifng	*Hif-2α$^{fl/fl}$*<br>*LysM$^{Cre/+}$ Hif-2α$^{fl/fl}$*<br>*LysM$^{Cre/Cre}$ Hif-2α$^{fl/fl}$*	1,04<br>1,11<br>0,79	0,12<br>0,31<br>0,55	7<br>6<br>3
	Hif-1α$^{fl/fl}$ × *Hif-2α$^{fl/fl}$*<br>*LysM$^{Cre/+}$ Hif-1α$^{fl/fl}$* × *Hif-2α$^{fl/fl}$*	0,9<br>0,81	0,29<br>0,20	7<br>7

Tnfa	*Hif-2α*$^{fl/fl}$<br>*LysM*$^{Cre/+}$ *Hif-2α*$^{fl/fl}$<br>*LysM*$^{Cre/Cre}$ *Hif-2α*$^{fl/fl}$	1,01<br>1,01<br>0,92	0,06<br>0,14<br>0,10	7<br>6<br>3
	Hif-1α$^{fl/fl}$ × *Hif-2α*$^{fl/fl}$<br>*LysM*$^{Cre/+}$ *Hif-1α*$^{fl/fl}$ × *Hif-2α*$^{fl/fl}$	0,98<br>1,25	0,07<br>0,06	7<br>7
Il23a	*Hif-2α*$^{fl/fl}$<br>*LysM*$^{Cre/+}$ *Hif-2α*$^{fl/fl}$<br>*LysM*$^{Cre/Cre}$ *Hif-2α*$^{fl/fl}$	1,04<br>0,89<br>1,23	0,12<br>0,24<br>0,20	7<br>6<br>3
Il17a	*Hif-2α*$^{fl/fl}$<br>*LysM*$^{Cre/+}$ *Hif-2α*$^{fl/fl}$<br>*LysM*$^{Cre/Cre}$ *Hif-2α*$^{fl/fl}$	1,02<br>1,07<br>0,91	0,08<br>0,19<br>0,48	7<br>6<br>3
Il6	*Hif-2α*$^{fl/fl}$<br>*LysM*$^{Cre/+}$ *Hif-2α*$^{fl/fl}$<br>*LysM*$^{Cre/Cre}$ *Hif-2α*$^{fl/fl}$	1,02<br>0,90<br>0,68	0,10<br>0,20<br>0,47	7<br>6<br>3
Cxcl1	*Hif-2α*$^{fl/fl}$<br>*LysM*$^{Cre/+}$ *Hif-2α*$^{fl/fl}$<br>*LysM*$^{Cre/Cre}$ *Hif-2α*$^{fl/fl}$	1,02<br>0,70<br>1,16	0,08<br>0,10<br>0,35	7<br>6<br>3
Il10	*Hif-2α*$^{fl/fl}$<br>*LysM*$^{Cre/+}$ *Hif-2α*$^{fl/fl}$<br>*LysM*$^{Cre/Cre}$ *Hif-2α*$^{fl/fl}$	1,018<br>1,10<br>1,09	0,08<br>0,22<br>0,71	7<br>6<br>3
	Hif-1α$^{fl/fl}$ × *Hif-2α*$^{fl/fl}$<br>*LysM*$^{Cre/+}$ *Hif-1α*$^{fl/fl}$ × *Hif-2α*$^{fl/fl}$	1,07<br>1,21	0,15<br>0,20	7<br>7
Tgfb1	*Hif-2α*$^{fl/fl}$<br>*LysM*$^{Cre/+}$ *Hif-2α*$^{fl/fl}$<br>*LysM*$^{Cre/Cre}$ *Hif-2α*$^{fl/fl}$	1,00<br>1,12<br>0,85	0,03<br>0,18<br>0,24	7<br>6<br>3
Arg1	*Hif-2α*$^{fl/fl}$<br>*LysM*$^{Cre/+}$ *Hif-2α*$^{fl/fl}$<br>*LysM*$^{Cre/Cre}$ *Hif-2α*$^{fl/fl}$	1,01<br>1,07<br>1,09	0,07<br>0,23<br>0,43	7<br>6<br>3
	Hif-1α$^{fl/fl}$ × *Hif-2α*$^{fl/fl}$<br>*LysM*$^{Cre/+}$ *Hif-1α*$^{fl/fl}$ × *Hif-2α*$^{fl/fl}$	0,93<br>1,16	0,19<br>0,21	7<br>7
